# Simultaneous detection of genomic imbalance in patients receiving preimplantation genetic testing for monogenic diseases (PGT-M)

**DOI:** 10.3389/fgene.2022.976131

**Published:** 2022-09-29

**Authors:** Lin Yang, Yan Xu, Jun Xia, Huijuan Yan, Chenhui Ding, Qianyu Shi, Yujing Wu, Ping Liu, Jiafu Pan, Yanhong Zeng, Yanyan Zhang, Fang Chen, Hui Jiang, Yanwen Xu, Wei Li, Canquan Zhou, Ya Gao

**Affiliations:** ^1^ College of Life Sciences, University of Chinese Academy of Sciences, Beijing, China; ^2^ BGI-Shenzhen, Shenzhen, China; ^3^ Reproductive Medicine Center, The First Affiliated Hospital, Sun Yat-sen University, Guangzhou, China; ^4^ Guangdong Provincial Key Laboratory of Reproductive Medicine, The First Affiliated Hospital, Sun Yat-sen University, Guangzhou, China; ^5^ BGI Genomics, BGI-Shenzhen, Shenzhen, China; ^6^ Hebei Industrial Technology Research Institute of Genomics in Maternal and Child Health, Shijiazhuang, China; ^7^ Shenzhen Engineering Laboratory for Birth Defects Screening, Shenzhen, China

**Keywords:** monogenic disease, chromosome abnormality, IVF, PGT-A/M, TAGs-seq

## Abstract

**Background:** Preimplantation genetic test for monogenic disorders (PGT-M) has been used to select genetic disease-free embryos for implantation during *in vitro* fertilization (IVF) treatment. However, embryos tested by PGT-M have risks of harboring chromosomal aneuploidy. Hence, a universal method to detect monogenic diseases and genomic imbalances is required.

**Methods:** Here, we report a novel PGT-A/M procedure allowing simultaneous detection of monogenic diseases and genomic imbalances in one experiment. Library was prepared in a special way that multiplex polymerase chain reaction (PCR) was integrated into the process of whole genome amplification. The resulting library was used for one-step low-pass whole genome sequencing (WGS) and high-depth target enrichment sequencing (TES).

**Results:** The TAGs-seq PGT-A/M was first validated with genomic DNA (gDNA) and the multiple displacement amplification (MDA) products of a cell line. Over 90% of sequencing reads covered the whole-genome region with around 0.3–0.4 × depth, while around 5.4%–7.3% of reads covered target genes with >10000 × depth. Then, for clinical validation, 54 embryos from 8 women receiving PGT-M of β-thalassemia were tested by the TAGs-seq PGT-A/M. In each embryo, an average of 20.0 million reads with 0.3 × depth of the whole-genome region was analyzed for genomic imbalance, while an average of 0.9 million reads with 11260.0 × depth of the target gene *HBB* were analyzed for β-thalassemia. Eventually, 18 embryos were identified with genomic imbalance with 81.1% consistency to karyomapping results. 10 embryos contained β-thalassemia with 100% consistency to conventional PGT-M method.

**Conclusion:** TAGs-seq PGT-A/M simultaneously detected genomic imbalance and monogenic disease in embryos without dramatic increase of sequencing data output.

## Introduction

There are currently more than 7,000 known monogenic diseases, and each person carries a few pathogenic mutations (Bell et al., 2011; He et al., 2017; Vaz-de-Macedo and Harper 2017). Parents carrying disease causative mutations are likely to transmit to their next generation and cause monogenic diseases in children. In 1990s, preimplantation genetic testing for monogenic diseases (PGT-M), known as preimplantation genetic diagnosis by then, has been developed to prevent the vertical transmitting of the causative mutations from parents to children by transplanting embryos free of diseases during the *in vitro* fertilization (IVF) treatment (Handyside et al., 1990; Huang et al., 2015). Since then, over 22,740 cycles of PGT-M have been tested to detect more than 190 different monogenic disorders (Harper et al., 2012; Dreesen et al., 2014; De Rycke et al., 2015; De Rycke et al., 2017; Coonen et al., 2020; van Montfoort et al., 2021).

The clinical utility of PGT-M in detecting monogenic disease has been well established (Van Rij et al., 2012; Drusedau, et al., 2013; Group et al., 2020). However, the embryos tested by PGT-M have substantial risks of chromosomal aneuploidy compared to women of natural conception (Gianaroli et al., 2003; Chang et al., 2016; Sciorio et al., 2020). A meta-analysis calculated the weighted aneuploidy rate in embryos, ranging from 20.7% to 100% relative to the maternal age (Franasiak et al., 2014). Li et al. (2018) observed approximately 26.5% of the normal or nonpathogenic blastocysts diagnosed by PGT-M were aneuploidy, although the population had a relatively young maternal age (31.9 ± 4.1 year). Kara et al. reported an even higher aneuploidy rate (33.5%) among the unaffected blastocysts diagnosed by PGT-M despite a young maternal age (32.4 ± 5.9 year). They also demonstrated that 53.2% of patients had ≥1 blastocyst that was free of the monogenic disorder but aneuploid, and concluded that a concurrent test of PGT-A and PGT-M would provide valuable information for embryo selection (Goldman et al., 2016).

Several PGT-A/M procedures have been reported to detect chromosome aneuploidies while testing for monogenic diseases. Konstantinidis et al. (2015) determined the monogenic disease and chromosome aneuploidy in embryos using different aliquots of the same multiple displacement amplification (MDA) products through karyomapping method. However, the monogenic disease and chromosome aneuploidy were tested by separate procedures in each embryo, and the pathogenic mutations could not be detected directly. Yan et al. (2015) developed a combined procedure named the mutated allele revealed by sequencing with aneuploidy and linkage analyses (MARSALA) to detect genomic imbalances and monogenic diseases simultaneously. However, this procedure required separate steps of low-coverage genome sequencing (0.1 × coverage) and low-coverage targeted sequencing (2 × coverage). Moreover, the MARSALA method required prior knowledge of mutation locations for primer design, and thus must be tailored in different clinical situations. Backenroth et al. reported an all-in-one PGT-A/M procedure (Haploseeks) that employed haplotype predication using low-coverage genome sequencing of blastocyst DNA by next-generation sequencing (NGS) (Backenroth et al., 2019). However, their method required precise processing of the PGT-M couple and a first-degree family member with SNP array to establish accurate haplotypes. Besides, like all other methods based on haplotype deduction, the Haploseeks method could have problems when cross recombination events occur near the mutation location. Recently, Mai et al. (2020) reported a combined procedure of PGT-M (β-thalassemia) plus PGT-A using the degenerate oligomer primer polymerase chain reaction (PCR) method. However, the primers used for *HBB* amplification was limited to mutations and single nucleotide polymorphisms (SNPs) specific for Vietnamese population. A generic NGS based OnePGT solution and a NGS based single-cell genotyping-by-sequencing method were reported recently (Masset et al., 2019; Masset et al., 2022). However, information obtained from additional family members is still required, and the risk of incorrect haplotyping still exists.

To overcome the abovementioned limitations, we developed a new NGS-based PGT-A/M method to amplify the genome in a way that the low-pass whole genome sequencing (WGS) and high-depth target enrichment sequencing (TES) can be integrated into a universal experiment. This method, also referred as the Targeted And Genome-wide Simultaneous Sequencing (TAGs-seq) (Yang et al., 2021), thus allows the simultaneous detection of embryo genomic imbalances and monogenic disease using the low-pass WGS reads and high-depth TES reads, respectively. The analytic utility and clinical efficacy of this TAGs-seq PGT-A/M was validated with cell line DNA and retrospective clinical samples to simultaneously detect β-thalassemia and genomic imbalances.

## Materials and methods

### Study design

Simultaneous detection of β-thalassemia and genomic imbalances by TAGs-seq based PGT was firstly validated for its analytic utility using the genomics DNA (gDNA) and MDA product of a cell line. Then, the approach was validated with the clinical samples retrospectively collected from trio families receiving routine PGT-M of β-thalassemia at the First Affiliated Hospital of Sun-Yet Sen University, China. Previously, routine clinical service of PGT-M was provided either by the SNP array based Karyomapping (Illumina, Inc.) or NGS based method (Jabrehoo, China) independent of this study. The TAGs-seq results of this study were not used for clinical purposes. The previous clinical testing results were undisclosed to the laboratory personnel until the TAGs-seq analysis done.

### Targeted and genome-wide simultaneous sequencing based preimplantation genetic testing

Generally, 1ug gDNA or MDA products were fragmented, end-repaired and ligated with DNBSEQ-G400 universal adapters (Supplementary Table S1). The ligated products were subjected to two rounds of PCR using a KAPA 2G Fast Multiplex PCR kit (Kapa Biosystems, United States). The first round of PCR was performed using a first pool of gene specific primers (GSP pool1) and a universal primer1 (UP1) complementary to the universal adapters. In brief, 20 μl ligated products, 25 μl 2 × KAPA PCR master mix, 2.5 μl 10 μM UP1 [5′-TGTGAGCCAAGGAGTTG-3′], and 2.5 μl GSP pool1 (final concentrations of each primer was 0.5 nmol/L) were added to a PCR tube and amplified in a S1000 Thermal Cycler (Bio-Rad, United States) with the following program: 98°C for 2 min; 20 cycles of 98°C for 10 s, 60°C for 2 min, 72°C for 30 s; 72°C for 5 min. After cleanup the amplification products, a second round of PCR was performed in another 50-μl reaction containing 17.5 μl first-round amplification products, 2.5 μl 10 μM UP1 [5′-TGTGAGCCAAGGAGTTG-3′], 2.5 μl 10 μM universal primer2 (UP2) [5′-/5Phos/GAACGACATGGCTACGATCCGACTT-3′] and a second pool of gene specific nested primers (GSP pool2, final concentrations of each primer was 0.25 nmol/L) following the reaction conditions: 98°C for 2 min; 10 cycles of 98°C for 10 s, 60°C for 2 min, 72°C for 30 s; 72°C for 5 min. After cleanup, the final amplification products were quantified by Qubit using an ssDNA HS Assay kit (Invitrogen, United States). The final amplification products contained the reads mixture of genome-wide and targeted amplicons, which were then normalized and processed for circularization and sequencing on a DNBSEQ-G400 platform (MGI, China) under SE50 sequencing strategy. In this study, a total of 85 TAGs-seq primer pairs were designed to amplify the HBB gene region and 65 SNPs with high minor allele frequency (MAF>0.35) which distribute in 1 Mb flanking region of HBB gene (Supplementary Figure S1; Supplementary Table S2). PCR primers specific for HBB gene and flanking SNPs were designed using Primer3.

### Analytic validation in cell line

The analytic utility of detecting single nucleotide variants (SNVs) and copy number variants (CNVs) by the TAGs-seq based PGT was performed using a fibroblast cell line (catalog number GM03918, Coriell Institute) which contains a known deletion (46,XX,del(2)(q31q32).arr 2q31.1q32.3(174584215–193944973) × 1). Bulk cells gDNA and few cells MDA product of the cell line were prepared, and tested by ∼30× WGS and TAGs-seq for result comparison. For bulk cells gDNA preparation, we used DMEM medium (Sigma-Aldrich) supplemented with 15% FBS (Gibco, United States) to culture cells to 10^7^. Then, we extracted gDNA from cells using a QIAamp DNA Mini Kit (Qiagen, Germany) following the manufacturer’s instructions. For MDA product preparation, 3–5 cells were sorted to a 200 μl PCR-tube from the cultured solution by FACSJazz Flow cytometry (BD, United States). We used REPLI-g Single Cell Kit (Qiagen, Germany) to amplify the whole genome of sorted cells following the manufacturer’s instructions. Briefly, 3 μl buffer D2 was added in the PCR-tube which contains 3–5 cells in 4 μl PBS and followed by the incubation at 65°C for 10 min. After the incubation, 3 μl stop solution was added to stop the reaction. Then, a WGA master mix containing 10 μl nuclease-free water, 29 μl REPLI-g sc Reaction Buffer and 2 μl EPLI-g sc DNA Polymerase was added to the cell lysate followed by the isothermal amplification at 30°C for 8 h and inactivation at 65°C for 3 min.

### Clinical validation of embryos

Eight couples who have been receiving intracytoplasmic sperm injection (ICSI) treatment and traditional PGT-M of β-thalassemia were retrospectively selected to validate the performance of the TAGs-seq based PGT. The eight couples, from 31 to 41 years old (Supplementary Table S3), each carried heterozygous mutations in *HBB* pathogenic loci, including CD41-42 (-CTTT), CD17 (A > T), IVS-2-654 (C > T), and each of their first child was a homozygous affected fetus. Out of which only one sample from one couple underwent the third PGT cycles, the rest samples were in the first cycle.

After ICSI, embryos were cultured for 5 days until they reached the blastocyst stage. Embryo biopsy was performed at the blastocyst stage using laser technology. A few (6–8) trophectoderm cells were biopsied from one embryo with accepted quality and morphology. After biopsy, all blastocysts were vitrified and cryopreserved immediately, and a deferred transfer was performed after PGT. The biopsied cells were transferred into individual sterile 200 μl PCR tubes, which contained 4 μl PBS. Single-cell whole genome amplification was performed by MDA using the REPLI-g SC kit (Qiagen, Germany) according to the manufacturer’s instructions. In addition, 5 ml peripheral blood collected from each couple and their proband offspring was used for gDNA extraction using a QIAamp DNA Mini Kit (Qiagen, Germany) according to the manufacturer’s instructions. In clinical practice, six families have received PGT-M by Karyomapping based Illumina HumanKaryomap-12 BeadChip (Illumina, Inc), and two families have received PGT-M by NGS-based methods (Jabrehoo, China). For clinical validation in this study, TAGs-seq was conducted in the eight families with the gDNA of family members and the MDA products of the embryos. The TAGs-seq results were compared with clinical PGT-M results (Supplementary Table S3).

### Whole genome sequencing library preparation and sequencing.

WGS libraries were prepared using MGIEasy FS DNA Library Prep Kit (MGI, China) following the manufacturer’s instructions (Zhang et al. 2013). Briefly, 1ug of gDNA or MDA product was fragmented into a length distribution of 100–1,000 bp with a peak of 350 bp. Then the fragments were incubated with magnetic beads which preferentially bind to specific DNA fragments size (200–700 bp), followed by the elusion and recovery of fragments with desired sizes. Then, the fragmentation product was transferred to a separate tube for end-repairing, A-tailing and ligation. The ligation product was amplified for seven cycles using primers complementary to the ligated adapters. Ligation products were then normalized and processed for circularization (Xu et al. 2019). Briefly, ligation products were heat-denatured at 95°C for 3 min to make a single strand DNA circle (ssDNA circle), which were then mixed reagents of MGIEasy FS DNA Library Prep Kit (MGI, China) and incubated at 37°C for 30 min to complete the circularization. The resulting ssDNA circle was then used to generate DNA nanoballs (DNBs) by rolling circle amplification (RCA) (Drmanac et al. 2010). After RCA and DNBs formation, the final product was measured by Qubit using an ssDNA HS Assay kit (Invitrogen, United States) and loaded on a DNBSEQ-G400 platform (MGI, China) by a DNB manual loader to undergo ∼30× WGS following a PE150 sequencing strategy. Raw fastq files were generated as described previously using the zebracall software (v2.0) provided by the manufacturer (Huang et al. 2017).

### Analysis of β-thalassemia and genomic imbalances.

After TAGs-seq, the low-pass WGS reads were used to analyze genomic imbalances, while the high-depth gene-specific TES reads were used to analyze the mutations of β-thalassemia and nearby SNPs. TAGs-seq raw data were trimmed to remove adapter sequences and filtered by SOAPnuke (v1.5.6), and then mapped to the human reference genome (hg19) using the BWA tool (v0.7.15). PE reads longer than 500 nt and non-unique alignments reads were removed. On-target reads mapped to the SNPs, HBB gene and flanking regions were used for monogenic analysis. The off-target reads mapped to other regions of genome were used for chromosomal analysis.

For monogenic analysis, both direct genotyping and linkage analysis were used to determine the *HBB* mutations to avoid the influence of allelic drop-out (ADO) and preferential amplification. Direct genotyping exploited reads uniquely mapped to *HBB* with the sequencing depth greater than 100×. The bam file of on-target reads was sorted by Samtools (v1.9) followed by realignment using GATK toolkit (v3.30). iTools (v0.19) was used to calculate the four-base depth of target position. The allele was determined as homozygous only when the minor allele ratio was less than 5%. In the other hand, linkage analysis used 65 SNPs to establish the haplotype of the *HBB* gene. In each family, the SNPs heterozygous in at least one parent but homozygous in proband were defined as informative SNPs. At least 10 informative SNPs were required to infer the *HBB* genotype based on the haplotype analysis according to the Mendel’s Law (Mai et al., 2021).

Chromosomal analysis was performed according to the method described previously (Jiang et al., 2012). The reference genome (GRCh37, UCSC release hg19) was divided into sliding windows with 50 nt simulated reads and mapped back to the origin reference genome with a maximum of two mismatches. Among the 100 K simulated unique mapped reads in continuous windows, we allowed 20 K overlapping reads to exist. The GC content of each window was calculated and used for the GC-bias correction. The normalized depth ratio (NDR) is the unique mapped non-duplication reads of each window divided by the total average unique mapped non-duplication reads, which was used to calculate the coverage and evaluate the reproducibility and uniformity (Chen et al., 2018). We defined the copy ratio as the value after merging and averaging the NDR of each ten windows. A binary segmentation algorithm for CNV breakpoints identification, and dynamic threshold determination for a final signal filtering, was established to identify CNVs (Zhang et al., 2013). We calculated the normalized depth ratio of each chromosome according to the NDR value of the chromosome, and determined aneuploidy according to the variation length and depth fluctuation range (the normal ratio of the normalized depth value ranges from 0.7 to 1.3). The CNVs larger than 4M would be reported under TAGs-seq test. We do not detect mosaicism for chromosome copy number variations. All the results were visualized by digital karyotypes for better presentation

### Statistics

Data are presented as the mean ± SD and differences are considered statistically significant at *p* < 0.05 using Student’s *t*-test. For the cell line data, at least three independent cells experiments were performed. *p < 0.05, **p < 0.01. N.S means not significant. Statistical analyses were performed using R v4.0.2.

## Results

### Establishing targeted and genome-wide simultaneous sequencing preimplantation genetic testing-A/M using cell line DNA

**FIGURE 1:**
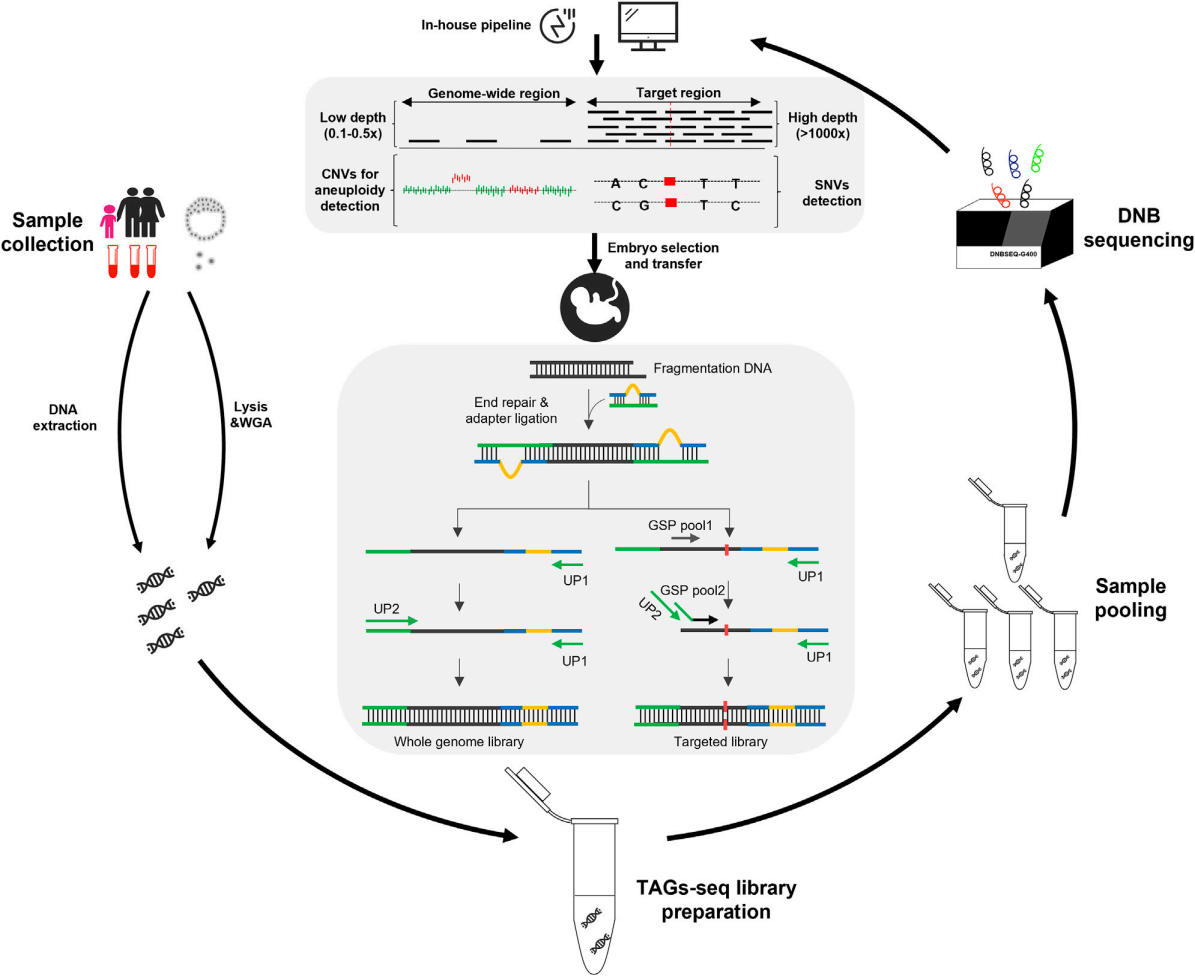
Overview of the TAGs-seq workflow for PGT-A and PGT-M. The gDNA products were extracted from the blood samples of the trio family, while the MDA product was prepared from a few (6–8) trophectoderm cells of an embryo. Then the TAGs-seq library preparation and pooling were performed, followed by the sequencing on DNBSEQ-G400 platform. The monogenic disorders and genomic imbalances were analyzed by an in-house pipeline. Finally, a healthy embryo was selected to transfer according to the result of PGT-A/M.

The TAGs-seq PGT-A/M is featured with an optimized library preparation process that can concordantly amply the genome-wide region at a low sequencing depth and genome target regions at an ultra-high sequencing depth (Figure 1). The sequencing reads of genome-wide region (WGS reads) can then be used to analyze genomic imbalance such as aneuploidy and CNV, whereas the sequencing reads of target regions (TES reads) can be used to analyze SNVs.

**FIGURE 2:**
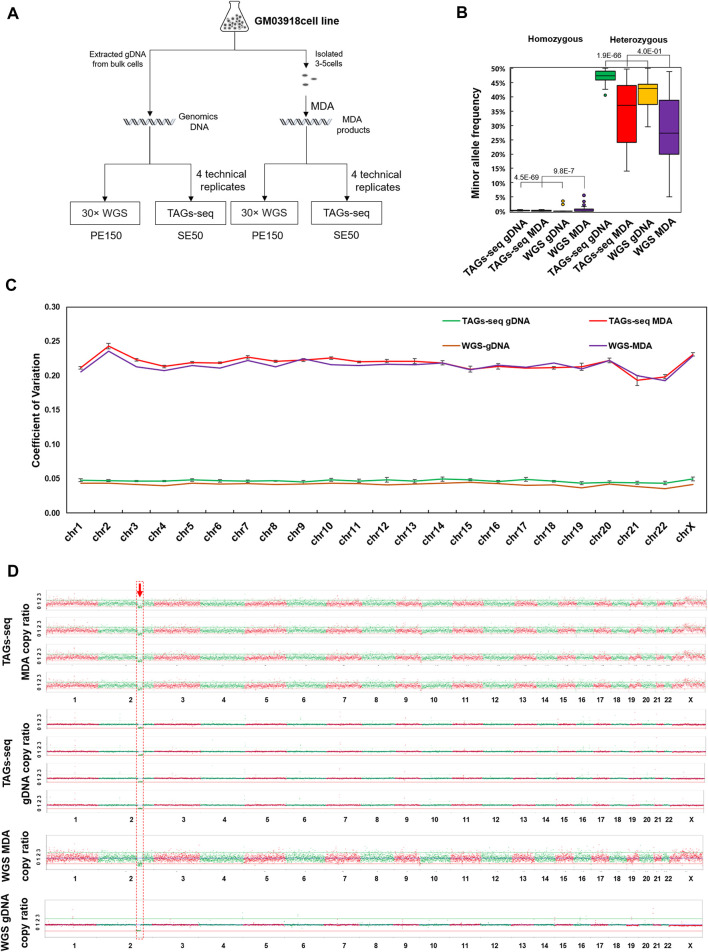
The performance of TAGs-seq in GM03918 cell line. **(A)** Experiment design of the TAGs-seq in GM03918 cell line. Bulk cells gDNA and multiple displacement amplification (MDA) product of the 3-5 isolated cells were prepared, which were then tested by ∼30× whole genome sequencing (WGS) and 0.1–0.5 × TAGs-seq (4 technical replicates), respectively. **(B)** Distribution of MAR of homozygous and heterozygous sites in gDNA and MDA products by the TAGs-seq and WGS. We define homozygous site which MAR lower than 10% and heterozygous sites which MAR equal or higher than 10%. MAR ratio was defined as the ratio of mean MAR of TAGs-seq data over mean MAR of ∼30xWGS data. **(C)** Coefficient of variation of copy ratio on all chromosomes. The interquartile range (IQR) of the copy ratio was quantified, and the data between Q1−1.5 × IQR and Q3+1.5 × IQR were selected whereas the outliers were excluded. **(D)** Copy ratio of TAGs-seq and WGS in gDNA and MDA products at the low sequencing depth (0.1×) of NGS, normalized to 9 million raw reads per sample.

Using the gDNA of bulk cells and MDA products of 3-5 cells, one sample of 30× ordinary WGS and four replicates of TAGs-seq were compared (Figure 2A). The 30× ordinary WGS of bulk cells gDNA generated 296.4 million PE150 reads with a mean sequencing depth of 30.3× and whole genome coverage of 99.1%, while the 30× ordinary WGS of the MDA product generated 305.5 million PE150 reads with a mean sequencing depth of 31.6× and whole genome coverage of 98.5% (Supplementary Table S4). The TAGs-seq of bulk cells gDNA generated 9.61–20.6 million (mean 16.2 million) SE50 reads in each replicate, which contained 92.5%–95.8% (mean 94.1% ± 1.1%) of WGS reads with the sequencing depth of 0.15-0.31× (mean 0.25 ± 0.06), and 3.7%–6.8% (mean 5.4% ± 1.1%) of TES reads mapped to the *HBB* gene and flanking regions with the sequencing depth of 7496.4–12394.4× (mean 9535.9 ± 2020.6×) in each replicate (Supplementary Table S4). Similarly, the TAGs-seq of MDA products generated 20.9–32.0 million (mean 28.3 million) SE50 reads in each replicate, which contained 91.1%–95.2% (mean 92.4% ± 1.6%) of WGS reads with the sequencing depth of 0.31–0.51× (mean 0.43 ± 0.07), and 4.5%–8.6% (mean 7.3% ± 1.7%) of TES reads mapped to the *HBB* gene and flanking region with the sequencing depth of 16377.7–28328.6× (mean 22974.6 ± 4865.3×) in each replicate (Supplementary Table S4).

### Validation of single nucleotide variants analysis by TAGs-seq in cell line

For the TAGs-seq TES reads in bulk cells gDNA, all 85 primer pairs targeting HBB gene and flanking SNPs had the amplicon on-target rate higher than 97.6% (mean 97.6% ± 0.3%). In comparison, the TAGs-seq TES reads in MDA products had 69 primer pairs of on-target rate higher than 97.0% (mean 97.0% ± 0.3%) (Supplementary Table S4; Supplementary Figure S1A,B). Yet, the coverage of 98.8% of amplicons varied within tenfold of the average level in both gDNA and MDA, suggesting an even coverage pattern in each group (Supplementary Figure S2). Owing to its high sequencing depth at the target region, TAGs-seq using both bulk cell gDNA and MDA products showed lower allelic bias of SNPs genotyping than 30× ordinary WGS (Figure 2B).

Using the minor allele ratio (MAR) of 10% as the cut-off value to differentiate homozygous from heterozygous SNPs, a total of 39 homozygous SNPs and 26 heterozygous SNPs at upstream and downstream of *HBB* gene were identified in both gDNA and MDA products using the TES reads of TAGs-seq, which were fully consistent with 30× ordinary WGS in gDNA (Supplementary Figure S3). Noteworthily, genotyping of two SNPs (rs10742372, and rs11040512) using 30× ordinary WGS of MDA products showed discrepant results of homozygous SNPs by showing the MAR of 8.6% (35×) and 5.0% (40×), respectively (arrows in Supplementary Figure S3). In contrast, the rs10742372 and rs11040512 were identified as heterozygous SNPs in TAGs-seq using MDA products, showing the mean MAR of 15.7% ± 2.7% and 16.0% ± 2.6%, and the mean sequencing depth of 37405.3 ± 8077.6× and 37405 ± 8077.6× in four replicates, respectively (Supplementary Table S5). The incorrect genotyping of rs10742372 and rs11040512 in 30× ordinary WGS of MDA product might be caused by preferential amplification or amplification failure due to relatively lower sequencing depth.

A total of 11 β-thalassemia associating hotspot pathogenic loci (−29(A>G), −28 (A>G), CD17 (A>T), β^E^ (CD26 G>A), IVS-1-1(G>T), IVS-1-5(G>C), CD27-28(+T), CD41-42 (-CTTT), CD43 (G>T), CD71-72(+A), IVS-2- 654(C>T)) in Southern China (Chen et al., 2010) were analyzed using TAGs-seq TES reads of gDNA and MDA products. No amplification failure or ADO were observed. The mean sequencing depths of the 11 loci in gDNA and MDA products were 8105.5 ± 3540.1× and 9111.7 ± 4310.0×, respectively. All loci (*n* = 88) were wild-type, as expected, and fully matched the counterpart results of 30× ordinary WGS in gDNA and MDA products (Supplementary Table S5). Thus, the sensitivity and specificity of TAGs-seq in detecting SNPs and pathogenic loci were 100% (94.48%–100.00%, 95%CI) and 100% (95.26%–100.00%, 95%CI), respectively.

### Validation of chromosomal analysis by targeted and genome-wide simultaneous sequencing in cell line

The TAGs-seq generated 0.15×−0.51× WGS reads evenly distributing on 23 pairs of chromosomes in both gDNA and MDA products (Figure 2C). The coefficient of variance (CV) of copy ratio of each window on each chromosome between TAGs-seq and ordinary 30×WGS in MDA products showed no statistical difference (*p* = 0.28). The CV of copy ratio between TAGs-seq and ordinary 30 × WGS in gDNA showed significant statistical difference (*p* = 1.3E-10), nevertheless, both copy ratio CV of TAGs-seq and ordinary 30 × WGS are slight (Figure 2C). In all replicates, a known 19.4M deletion (46,XX,del(2)(q31q32)) of the tested cell line was steadily detected (Figure 2D). No chromosomal aneuploidy was detected with the 0.15–0.51× WGS reads of TAGs-seq, as expected. All results were consistent with 30× ordinary WGS results. Thus, the WGS reads of TAGs-seq in both gDNA and MDA products achieved comparable performance with the counterpart 30× ordinary WGS.

### Clinical validation with embryos

**FIGURE 3:**
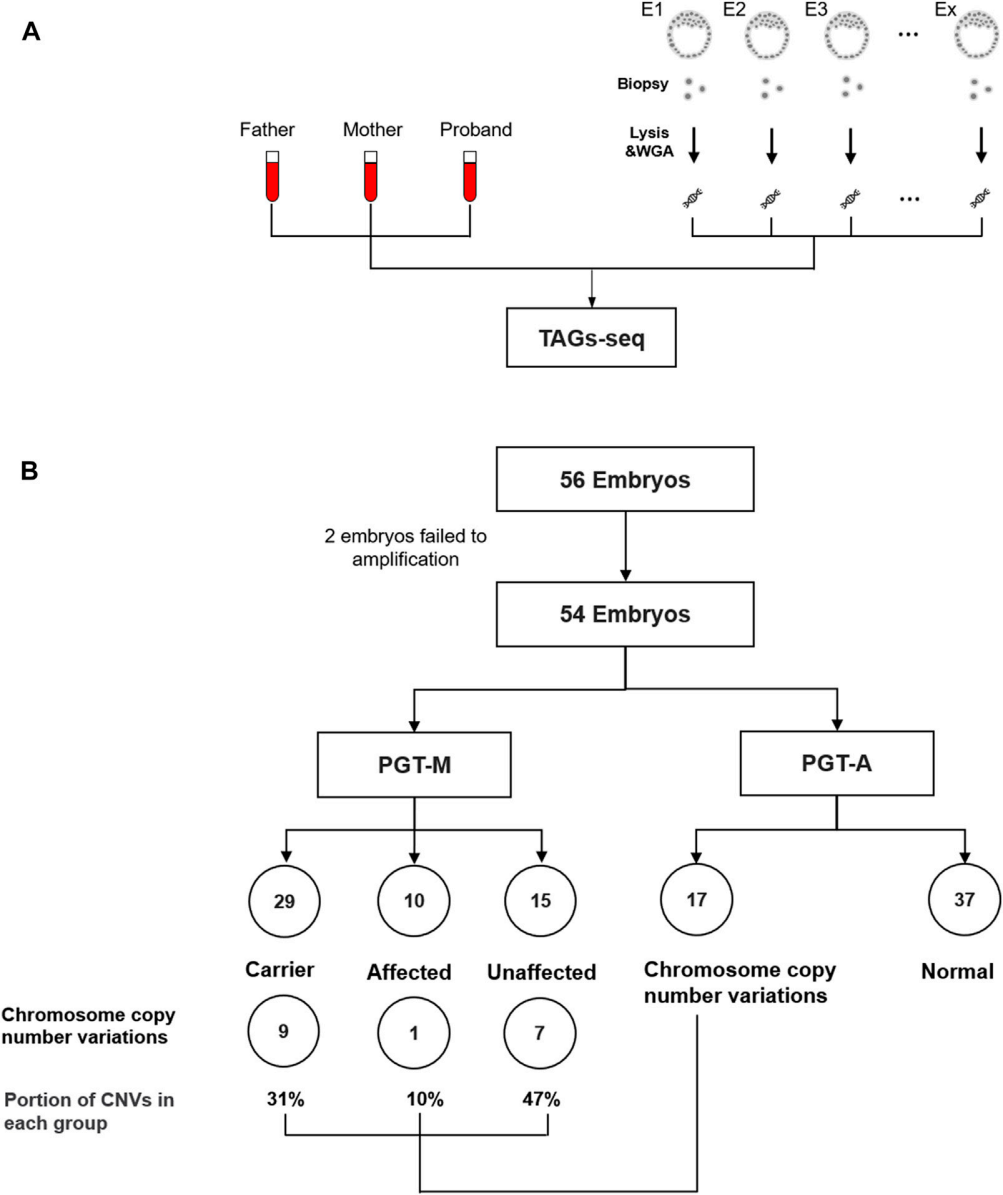
**(A)** TAGs-seq validation in clinical family sample. **(B)** Flowchart of analysis cohort of TAGs-seq samples. A total of 56 embryos were tested by TAGs-seq for PGT-A&M. The sequences data of 2 embryos were not sufficient quality due to the amplification failure. 29, 10 and 15 embryos were identified as carrier, affected and unaffected by PGT-M analysis. 17 and 37 embryos were identified with genomic imbalances and normal by the PGT-A analysis.

Eight families receiving conventional PGT-M for β-thalassemia were retrospectively selected to validate the TAGs-seq PGT-A/M. A total of 56 embryos were obtained, among which 2 could not receive MDA products and were excluded from the study. The failure of MDA may be due to loss of the cells during the experiment, probably missed by the tip. The MDA products of the remaining 54 embryos, plus 24 gDNA samples of the 8 trio families, were tested by the TAGs-seq PGT-A/M (Figure 3A; Supplementary Table S3). In each sample, the TAGs-seq PGT-A/M generated 6.5–39.8 million SE50 reads (mean 19.8 million) consisting of 89.5–99.6% (mean 95.5% ± 2.5%) of WGS reads with the sequencing depth of 0.11–0.65× (mean 0.31 ± 0.12×), and 0.4%–10.2% (mean 4.3% ± 2.4%) of TES reads mapped to the HBB coding areas and flanking regions with the sequencing depth of 603.3–28134.2 (mean 10782.7 ± 7783.8×) (Supplementary Table S6).

Using the high-depth TES reads of the TAGs-seq, β-thalassemia in 54 embryos was analyzed for PGT-M by direct genotyping of the 11 pathogenic alleles of *HBB* gene, as well as by linkage analysis using the *HBB* flanking SNPs. For the direct genotyping analysis, the mean reads covering the 11 pathogenic alleles were 21473.1 ± 20700.8 after removing low-quality reads. No ADO was observed. In total, 29 embryos (53.7%) carried one heterozygous pathogenic SNVs; 8 embryos (14.8%) carried compound heterozygous mutations; 2 embryos (3.7%) contained homozygous pathogenic SNVs; the remaining 15 embryos (27.8%) were determined as unaffected (Figure 3B). For the linkage analysis, a total of 65 *HBB* flanking SNPs in 78 samples (24 gDNA and 54 MDA products) were used. Among the total 5070 SNPs (65 × 78), only 2 could not be amplified due to ADO. The rest SNPs had the mean total reads of 10722, in which 94 SNPs (1.85%) were covered by less than 100 reads and thus were removed from the following linkage analysis. In the end, each embryo had 8.1 ± 1.2 informative SNPs for haplotype establishment (Supplementary Table S3). The linkage analysis of β-thalassemia in 54 embryos were fully consistent with direct genotyping except one embryo (Family 5 Embryo 7, F5E7), and the results were confirmed by conventional clinical PGT-M diagnosis (Karyomapping or NGS results) (Supplementary Table S3).

**FIGURE 4:**
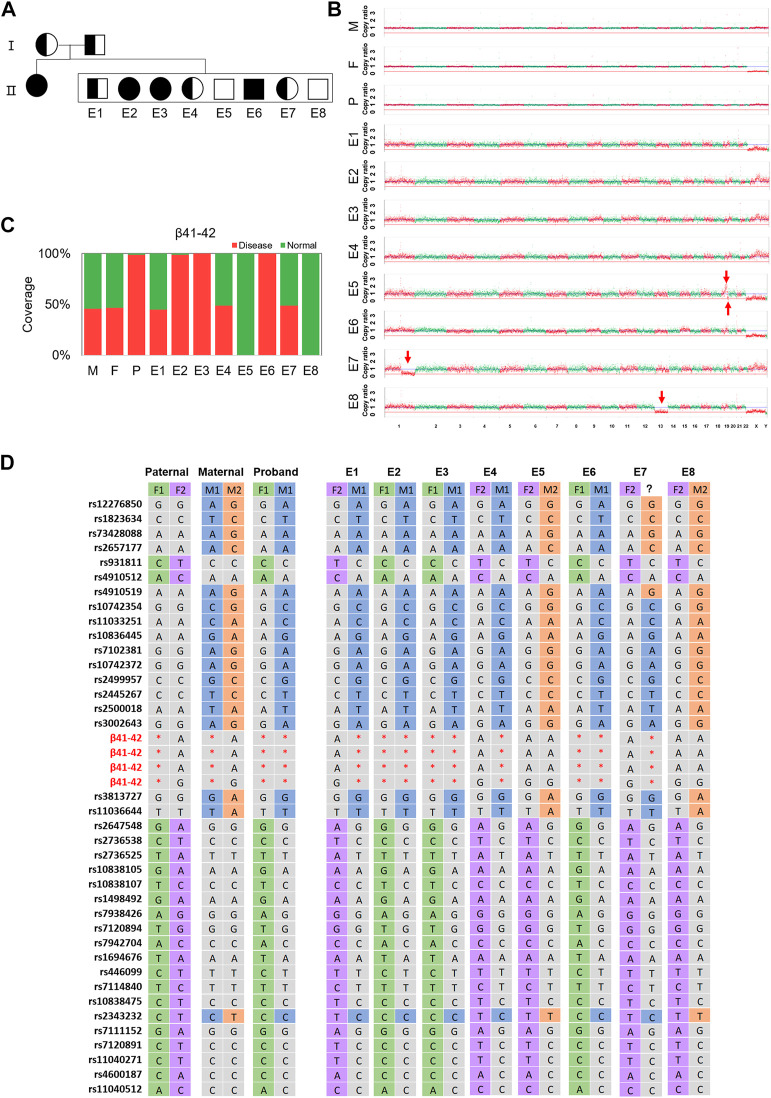
The results of TAGs-seq for family 5. **(A)** Pedigrees of the family with HBB. Filled symbols represent affected individuals; half-filled symbols represent carrier; open symbols represent normal individuals. Circles and squares indicate females and males, respectively. **(B)** CNVs of the embryos at the low sequencing depth (0.25–0.46×) of TAGs-seq. Significant genomic imbalance was identified in embryos E8 (monosomy 13); E1, E2, E3, E4 and E6 did not identify genomic imbalance; A deletion was found in E5 (19q13.33-19q13.43,8.55M) and E7 (1q21.1-1q44,103.70M); A duplication was found in E5 (19q13.11-19q13.33,17.51M); **(C)** Direct detection the targeted mutation site in the *HBB* gene of the father (F), the mother (M), the proband child(P) and 8 embryos (E1–E8) by TAGs-seq. E1, E4, E7 had one heterozygous mutated SNV (red), E2, E3, E6 had one homozygous mutated SNV and E5, 8 had no mutated SNV. The fraction of reads of the covered region shown in green is consistent with the reference genome. **(D)** Genotype of parents and embryos of the *HBB* gene plus 37 SNPs closely associated with the mutation. M1 and F1 were defined as maternal haplotype 1 and paternal haplotype 1, respectively; meanwhile M2 and F2 stands for the maternal haplotype 2 and paternal haplotype 2, respectively. Red star, mutation; green/purple/blue/orange/grey, no mutation.

Noteworthily, the linkage analysis of F5E7 revealed a recombination event near the *HBB* gene by showing two separate maternal haplotypes harboring 11 SNPs and 5 SNPs, respectively (Figure 4; Supplementary Table S3). This was confirmed by ∼30× WGS using the gDNA of the trio family and the MDA products of F5E7, showing the recombination occurring between 16q11.1-16q11.5 (Supplementary Figure S11).

Using the low-pass WGS reads of the TAGs-seq, genomic imbalance of 54 embryos were analyzed for PGT-A. In total, 17 embryos (31.5%) contained genomic imbalances, including 10 embryos (18.5%) of aneuploidies and 7 embryos (13.0%) of CNVs larger than 4 million base pairs (Supplementary Figures S4–S10; Supplementary Table S3). When compared the TAGs-seq results with conventional PGT-A (Karyomapping or NGS based method), 40 out of 51 (78.4%) embryos (three embryos not determined by conventional PGT-A) showed consistent results (Supplementary Table S3). The difference between the TAGs-seq PGT-A and conventional PGT-A is shown in red in Supplementary Table S3 with 2 aneuploidies and 9 CNVs (the minimum one is 4.61 M long).

Combining the results of TAGs-seq PGT-M and PGT-A, among 44 embryos free of β-thalassemia (carriers included), 16 (about 36.4%) contained chromosome aneuploidies or CNVs that were not appropriate for transfer.

## Discussion

We developed a universal method to obtain low-coverage WGS reads and ultra-high depth TES reads in a universal experiment, which allows the simultaneous detection of genomic imbalance and SNVs. Low-pass WGS by NGS (0.1–1×) has already been widely used in clinical practice to detect genomic imbalance, such as noninvasive prenatal testing (NIPT) or product of conception testing for genomic imbalance (Smith-Bindman and Miglioretti 2015; Dong et al. 2016). However, it requires a much deeper sequencing depth (typically > 90G data equivalent to ∼30× sequencing depth) to detect SNVs, which significantly increases the testing cost. Thus, using high-depth TES is popular in clinical scenario where detecting rare mutations is need, such as monogenic disease or cancer detection (Li et al. 2017; Wang et al. 2019). With the TAGs-seq approach, PGT-A and PGT-M were simultaneously performed with small data requirement (approximate 20 million of SE50 sequencing reads) and no complicated experiments. Owing to the feature that the majority of TAGs-seq reads were low-coverage WGS reads for chromosome analysis, and only 3%–5% of reads were high-depth TES data for SNVs analysis, TAGs-seq PGT-A/M had the benefits by providing an easy-working and affordable test when compared to previous PGT-A/M methods requiring multiple steps or different types of experiments (Yan et al. 2015; Mai et al. 2020).

A practical advantage of the TAGs-seq approach was that the concordant amplification of the whole genome and target regions had little mutual interference. The WGS reads, although with 0.1–0.5× coverage, had comparable chromosomal CV to ordinary 30× WGS. Meanwhile, the sequential amplification of using gene-specific primers allowed ultra-deep (9535–22974 × mean depth) sequencing of *HBB* gene and flanking SNPs for PGT-M. This resulted in much lower allelic bias of SNPs genotyping, and prevented the wrong genotyping results of two SNPs due to preference bias by 30× ordinary WGS in cell line MDA products. The ultra-deep sequencing also contributed to a low risk of ADO during SNPs genotyping, as only 2 out of 5070 SNPs had ADO in embryo MDA products. Eventually, the analytical sensitivity and specificity of TAGs-seq PGT-M/A was both 100% in detecting SNPs and pathogenic loci. The clinical sensitivity and specificity in detecting embryo genomic imbalance, especially CNVs by TAGs-seq PGT-A could not be defined because the conventional PGT-A results by Karyomapping or other NGS methods could not be used as the golden standards due to their own risk of incorrect results. It has been shown that Karyomapping using embryo MDA products might be prone to CNVs calling mistakes due to ADO or preference bias during the amplification (Dreesen et al., 2014). However, our TAGs-seq PGT-M/A approach showed high consistence with the conventional PGT-M/A results in clinical validation.

The TAGs-seq PGT-A/M also demonstrated a technical advantage that monogenic disease could be diagnosed by both direct genotyping and linkage analysis of bilateral SNPs. Previous PGT-M methods that detect SNVs by direct genotyping (Wiltonl et al., 2009; Dreesen et al., 2014) is vulnerable to ADO and amplification failure, which has been proven to be a major problem for PGT-M, especially when performing WGA by MDA (Wilton et al., 2009; Zimmerman et al., 2016). Furthermore, previous PGT-M methods employing linkage analysis such as short tandem repeat (STR) or Karyomapping may be affected by the small risk of homologous recombination close to the mutation loci (Dreesen et al., 2000). To address these issues, our method used TES reads of *HBB* coding regions and bilateral SNPs for direct genotyping and linkage analysis, respectively, and this double verification improved the accuracy and reliability of disease diagnosis. Unlike the Haploseek method which requires microarray testing in parents and low-pass sequencing in embryos to establish parental haplotypes (Backenroth et al., 2019), our method took a universal testing strategy in parents, proband, and embryos, thus reducing the difficulty of experiment operation and data integration.

Other minor benefits of TAGs-seq PGT-A/M also include 1) non-hotspot and *de novo* mutations, as well as CNVs on *HBB* gene, if any, may be detected since the entire *HBB* coding regions were amplified. Thus, unlike previous methods such as MARSALA(Yan et al., 2015), the prior knowledge of pathogenic information in proband and parents may not be prerequisites for TAGs-seq PGT-A/M; 2) TES primers could be easily added to the current TAGs-seq PGT-A/M procedure to expand the testing scope to other monogenic diseases. In future, an extended TAGs-seq PGT-A/M panel is likely to further reduce cost in clinical practice.

Noteworthily, in our study, we found that 16 embryos that were free of β-thalassemia had genomic imbalances, which potentially reduced the transferrable rate from 81.4% (44/54) to 51.9% (28/54). This would increase the risk of experiencing a cycle with no transferable embryos, particularly in women with advanced maternal age. Implanting chromosomally normal embryos has been thought to be beneficial to pregnant outcomes. However, recent evidence suggested that transferring blastocysts containing aneuploid cells could lead to healthy birth due to mechanisms of embryo self-rescue (Capalbo et al., 2021; Rito et al., 2021; Yang et al., 2021). In ordinary sub-fertile women receiving IVF treatment, Chen et al. recently proved that conventional IVF resulted in a cumulative live-birth rate that was noninferior to the rate with PGT-A, which provide the evidence against the use of PGT-A in routine clinical practice (Yan et al., 2021). However, providing PGT-A in patients receiving PGT-M may still be beneficial, which requires further evidence of its clinical utility. For future potential large-scale clinical validation, the TAGs-seq PGT-A/M approach provides a cost-effective and convenient choice.

Several limitations existed in this study. Firstly, clinical validation of the TAGs-seq PGT-A/M was performed with retrospective embryo samples with no birth outcomes available, thereby it was not possible to determine whether the PGT-A/M results would improve implantation and pregnant outcomes. Secondly, there was the lack of golden standards to verify the genomic imbalance results of TAGs-seq PGT-A using retrospectively collected embryos. Another limitation is that we only detected chromosome copy number variations and did not detect other chromosomal abnormalities, like translocations, inversions. Furthermore, we did not detect chromosomal mosaicism.

## Conclusion

The TAGs-seq PGT-A/M approach concordantly amplified the genome to generate low-coverage WGS reads for chromosome analysis and high-depth TES reads for SNVs analysis. In cell line gDNA and MDA products, the TAGs-seq PGT-A/M approach showed good analytic utility in detecting SNVs and CNVs. Using retrospectively collected embryos, the TAGs-seq PGT-A/M approach proved to accurately detect β-thalassemia by both direct genotyping and indirect linkage analysis, and simultaneously identified genomic imbalances with good consistence to conventional PGT-A/M. This proof-of-concept study demonstrated good potential for future clinical application with an extendable list of monogenic diseases.

## Data Availability

The data presented in the study are deposited in the CNSA repository, accession number CNP0002626.

## Supplementary material

SUPPLEMENTARY FIGURE S1 Primer design of TAGs-seq. **(A)**Schematic of the primer design of TAGs-seq, gene specific primer 1 and gene specific primer2 are designed at the same side and close to the target SNP or region. **(B)** Primers distribution of PGT-M for β-thalassemia detect, all primers distribute on 1 Mb flanking region of HBB gene.

SUPPLEMENTARY FIGURE S2 Primer performance of TAGs-seq.**(A,B)** The on-target rate of TAGs-seq in gDNA and MDA. **(C)** Normalized coverage relative to the mean coverage of covered total target.

SUPPLEMENTARY FIGURE S3 Compared the SNV detection of TAGs-seq with ∼30 × WGS in MDA and gDNA. rs10742372 and rs11040512 were shown below the red arrow. The detection of TAGs-seq in MDA products is more closely to the ∼30 × WGS in gDNA than ∼30 × WGS in MDA products.

SUPPLEMENTARY FIGURE S4 The results of TAGs-seq for family 1.**(A)** Pedigrees of the family with HBB. Filled symbols represent affected individuals; half-filled symbols represent carrier; open symbols represent normal individuals. Circles and squares indicate females and males, respectively. **(B)** CNVs of the embryos at the low sequencing depth (0.30–0.50×) of TAGs-seq. Significant genomic imbalance was identified in embryos E3(trisomy 1), E5(trisomy4), E6 (monosomy 16); E1, E2, and E4 did not identify genomic imbalance. **(C)** Direct detection the targeted mutation site in the HBB gene of the father (F), the mother (M), the proband child (P) and 6 embryos (E1–E6) by TAGs-seq. E1,3,5,6 had one heterozygous mutated SNV (red), E2 had one homozygous mutated SNV and E4 had no disease mutated SNV. The fraction of reads of the covered region shown in green is consistent with the reference genome.

SUPPLEMENTARY FIGURE S5 The results of TAGs-seq for family 2.**(A)** Pedigrees of the family with HBB. Filled symbols represent affected individuals; half-filled symbols represent carrier; open symbols represent normal individuals. Circles and squares indicate females and males, respectively. E1 was failed in amplification. **(B)** CNVs of the embryos at the low sequencing depth (0.28–0.42×) of TAGs-seq. Significant genomic imbalance was identified in embryos E2 (monosomy 16, monosomy 21), E4 (monosomy 21), E7 (monosomy 16), E8 (monosomy 16); E3, E5 and E6 did not identify genomic imbalance. **(C)** Direct detection the targeted mutation site in the HBB gene of the father (F), the mother (M), the proband child (P) and 7 embryos (E2–E8) by TAGs-seq. E4 had one heterozygous mutated SNV (red), E6 had two heterozygous mutated SNVs and E2, E3, E5, E7, E8 had no disease mutated SNV. The fraction of reads of the covered region shown in green is consistent with the reference genome.

SUPPLEMENTARY FIGURE S6 The results of TAGs-seq for family 3.**(A)** Pedigrees of the family with HBB. Filled symbols represent affected individuals; half-filled symbols represent carrier; open symbols represent normal individuals. Circles and squares indicate females and males, respectively. **(B)** CNVs of the embryos at the low sequencing depth (0.29–0.50×) of TAGs-seq. Significant genomic imbalance was identified in embryos E2 (trisomy X); A deletion found in E5 (9p24.3-9p21.3,21.69M); E1, E3 and E4 did not show any genomic imbalance. **(C)** Direct detection the targeted mutation site in the HBB gene of the father (F), the mother (M), the proband child (P) and 5 embryos (E1–E5) by TAGs-seq. E4 had one heterozygous mutated SNV (red), E1, E3 had one homozygous pathogenic SNV and E2, E5 had no pathogenic SNV. The fraction of reads of the covered region shown in green is consistent with the reference genome.

SUPPLEMENTARY FIGURE S7 The results of TAGs-seq for family 4.**(A)** Pedigrees of the family with HBB. Filled symbols represent affected individuals; half-filled symbols represent carrier; open symbols represent normal individuals. Circles and squares indicate females and males, respectively. **(B)** CNVs of the embryos at the low sequencing depth (0.32–0.50×) of TAGs-seq. Significant genomic imbalance was identified in embryos E2 (trisomy 16), E6 (trisomy 17, monosomy 10); E1, E3, E4 and E5 did not identify genomic imbalance. **(C)** Direct detection the targeted mutation site in the HBB gene of the father (F), the mother (M), the proband child (P) and 6 embryos (E1–E6) by TAGs-seq. E2, E3, E5 had one heterozygous mutated SNV (red), E4, E6 had one homozygous mutated SNV and E1 had no disease mutated SNV. The fraction of reads of the covered region shown in green is consistent with the reference genome.

SUPPLEMENTARY FIGURE S8 The results of TAGs-seq for family 6.**(A)** Pedigrees of the family with HBB. Filled symbols represent affected individuals; half-filled symbols represent carrier; open symbols represent normal individuals. Circles and squares indicate females and males, respectively. **(B)** CNVs of the embryos at the low sequencing depth (0.20–0.41×) of TAGs-seq. A deletion was found in E4 (14q24.3-14q32.33,33,35M); Other embryos did not identify genomic imbalance. **(C)** Direct detection the targeted mutation site in the HBB gene of the father (F), the mother (M), the proband child (P) and 6 embryos (E1–E6) by TAGs-seq. E1, E2, E3, E4, E5 had one heterozygous mutated SNV (red), E6 had two heterozygous mutated SNVs. The fraction of reads of the covered region shown in green is consistent with the reference genome.

SUPPLEMENTARY FIGURE S9 The results of TAGs-seq for family 8.**(A)** Pedigrees of the family with HBB. Filled symbols represent affected individuals; half-filled symbols represent carrier; open symbols represent normal individuals. Circles and squares indicate females and males, respectively. **(B)** CNVs of the embryos at the low sequencing depth (0.11–0.27×) of TAGs-seq. A duplication was found in E6 (1p36.32-1p35.3, 25.59M) and E7 (1q12-1q23.3, 20.11M), other embryos did not identify genomic imbalance. **(C)** Direct detection the targeted mutation site in the HBB gene of the father (F), the mother (M), the proband child (P) and 10 embryos (E1–E10) by TAGs-seq. E1, E2, E3, E5, E6, E7, E10 had one heterozygous mutated SNV (red) and E8 and E9 had no disease mutated SNV. The fraction of reads of the covered region shown in green is consistent with the reference genome.

SUPPLEMENTARY FIGURE S10 The results of TAGs-seq for family 9.**(A)** Pedigrees of the family with HBB. Filled symbols represent affected individuals; half-filled symbols represent carrier; open symbols represent normal individuals. Circles and squares indicate females and males, respectively. **(B)** CNVs of the embryos at the low sequencing depth (0.17–0.31×) of TAGs-seq. All embryos did not identify genomic imbalance. **(C)** Direct detection the targeted mutation site in the HBB gene of the father (F), the mother (M), the proband child (P) and 7 embryos (E1–E7) by TAGs-seq. E1 and E6 had one heterozygous mutated SNV (red) and E2, E3, E4, E5, E7 had no disease mutated SNV. The fraction of reads of the covered region shown in green is consistent with the reference genome.

SUPPLEMENTARY FIGURE S11 Haplotype phasing of Embryo 7 from family 5.**(A)** Haplotypes of chromosome 11. F/M represents haplotypes from father and haplotypes from mother, respectively. Blue lines and red lines represent that these sites are from of different homologous chromosomes of father (F1/F2); Green lines and orange lines represent that these sites are from homologous chromosomes of mother (M1/M2). **(B)** Haplotypes of HBB gene on chromosome 11. Purple lines represent the mutation site. It is in the block of haplotype M2.

SUPPLEMENTARY FIGURE S12 Visualization of sequence read using IGV. The y axis represents read coverage; the x axis represents reference position and their respective exons below. HBB gene and flank region with a high depth coverage (Purple arrow) and the WGS except target region with a low pass coverage (Red arrow).

SUPPLEMENTARY TABLE S4 The sequencing reads information for cell lines.

SUPPLEMENTARY TABLE S5 The variations information for PGT-M.

SUPPLEMENTARY TABLE S6 The sequencing reads information for embryos.

## References

[B1] Backenroth D. Zahdeh F. Kling Y. Peretz A. Rosen T. Kort D. (2019). Haploseek: A 24-hour all-in-one method for preimplantation genetic diagnosis (PGD) of monogenic disease and aneuploidy. Genet. Med. 21 (6), 1390–1399. 10.1038/s41436-018-0351-7 30449887

[B2] Bell C. J. Dinwiddie D. L. Miller N. A. Hateley S. L. Ganusova E. E. Mudge J. (2011). Carrier testing for severe childhood recessive diseases by next-generation sequencing. Sci. Transl. Med. 3 (65), 65ra4. 10.1126/scitranslmed.3001756 PMC3740116 21228398

[B3] Capalbo A. Poli M. Rienzi L. Girardi L. Patassini C. Fabiani M. (2021). Mosaic human preimplantation embryos and their developmental potential in a prospective, non-selection clinical trial. Am. J. Hum. Genet. 108 (12), 2238–2247. 10.1016/j.ajhg.2021.11.002 34798051 PMC8715143

[B4] Chang J. Boulet S. L. Jeng G. Flowers L. Kissin D. M. (2016). Outcomes of *in vitro* fertilization with preimplantation genetic diagnosis: An analysis of the United States assisted reproductive technology surveillance data, 2011-2012. Fertil. Steril. 105 (2), 394–400. 10.1016/j.fertnstert.2015.10.018 26551441 PMC5023328

[B5] Chen D. Zhen H. Qiu Y. Liu P. Zeng P. Xia J. (2018). Comparison of single cell sequencing data between two whole genome amplification methods on two sequencing platforms. Sci. Rep. 8 (1), 4963. 10.1038/s41598-018-23325-2 29563514 PMC5862989

[B6] Chen W. Zhang X. Shang X. Cai R. Li L. Zhou T. (2010). The molecular basis of beta-thalassemia intermedia in southern China: Genotypic heterogeneity and phenotypic diversity. BMC Med. Genet. 11, 31. 10.1186/1471-2350-11-31 20181291 PMC2845123

[B7] Coonen E. van Montfoort A. Carvalho F. Kokkali G. Moutou C. Rubio C. (2020). ESHRE PGT consortium data collection XVI-XVIII: Cycles from 2013 to 2015. Hum. Reprod. Open 2020 (4), hoaa043. hoaa043. 10.1093/hropen/hoaa043 33033756 PMC7532546

[B8] De Rycke M. Belva F. Goossens V. Moutou C. SenGupta S. B. Traeger-Synodinos J. (2015). ESHRE PGD consortium data collection XIII: Cycles from january to december 2010 with pregnancy follow-up to october 2011. Hum. Reprod. 30 (8), 1763–1789. 10.1093/humrep/dev122 26071418

[B9] De Rycke M. Goossens V. Kokkali G. Meijer-Hoogeveen M. Coonen E. Moutou C. (2017). ESHRE PGD consortium data collection XIV-XV: Cycles from january 2011 to december 2012 with pregnancy follow-up to october 2013. Hum. Reprod. 32 (10), 1974–1994. 10.1093/humrep/dex265 29117384

[B10] Dong Z. Zhang J. Hu P. Chen H. Xu J. Tian Q. (2016). Low-pass whole-genome sequencing in clinical cytogenetics: A validated approach. Genet. Med. 18 (9), 940–948. 10.1038/gim.2015.199 26820068

[B11] Dreesen J. C. Jacobs L. J. Bras M. Herbergs J. Dumoulin J. C. Geraedts J. P. (2000). Multiplex PCR of polymorphic markers flanking the CFTR gene; a general approach for preimplantation genetic diagnosis of cystic fibrosis. Mol. Hum. Reprod. 6 (5), 391–396. 10.1093/molehr/6.5.391 10775641

[B12] Dreesen J. Destouni A. Kourlaba G. Degn B. Mette W. C. Carvalho F. (2014). Evaluation of PCR-based preimplantation genetic diagnosis applied to monogenic diseases: A collaborative ESHRE PGD consortium study. Eur. J. Hum. Genet. 22 (8), 1012–1018. 10.1038/ejhg.2013.277 24301057 PMC4350594

[B13] Drmanac R. Sparks A. B. Callow M. J. Halpern A. L. Burns N. L. Kermani B. G. (2010). Human genome sequencing using unchained base reads on self-assembling DNA nanoarrays. Science 327 (5961), 78–81. 10.1126/science.1181498 19892942

[B14] Drusedau M. Dreesen J. C. Derks-Smeets I. Coonen E. van Golde R. van Echten-Arends J. (2013). PGD for hereditary breast and ovarian cancer: The route to universal tests for BRCA1 and BRCA2 mutation carriers. Eur. J. Hum. Genet. 21 (12), 1361–1368. 10.1038/ejhg.2013.50 23531862 PMC3831069

[B15] Franasiak J. M. Forman E. J. Hong K. H. Werner M. D. Upham K. M. Treff N. R. (2014). The nature of aneuploidy with increasing age of the female partner: A review of 15, 169 consecutive trophectoderm biopsies evaluated with comprehensive chromosomal screening. Fertil. Steril. 101 (3), 656–663. e651. 10.1016/j.fertnstert.2013.11.004 24355045

[B16] Gianaroli L. Magli M. C. Ferraretti A. P. Fortini D. Grieco N. (2003). Pronuclear morphology and chromosomal abnormalities as scoring criteria for embryo selection. Fertil. Steril. 80 (2), 341–349. 10.1016/s0015-0282(03)00596-x 12909497

[B17] Goldman K. N. Nazem T. Berkeley A. Palter S. Grifo J. A. (2016). Preimplantation genetic diagnosis (PGD) for monogenic disorders: The value of concurrent aneuploidy screening. J. Genet. Couns. 25 (6), 1327–1337. 10.1007/s10897-016-9975-4 27277129

[B18] Group E. P.-M. W. Carvalho F. Moutou C. Dimitriadou E. Dreesen J. Gimenez C. (2020). ESHRE PGT Consortium good practice recommendations for the detection of monogenic disorders. Hum. Reprod. Open 2020 (3), hoaa018. 10.1093/hropen/hoaa018 32500103 PMC7257022

[B19] Handyside A. H. Kontogianni E. H. Hardy K. Winston R. M. (1990). Pregnancies from biopsied human preimplantation embryos sexed by Y-specific DNA amplification. Nature 344 (6268), 768–770. 10.1038/344768a0 2330030

[B20] Harper J. C. Wilton L. Traeger-Synodinos J. Goossens V. Moutou C. SenGupta S. B. (2012). The ESHRE PGD consortium: 10 years of data collection. Hum. Reprod. Update 18 (3), 234–247. 10.1093/humupd/dmr052 22343781

[B21] He J. Song W. Yang J. Lu S. Yuan Y. Guo J. (2017). Next-generation sequencing improves thalassemia carrier screening among premarital adults in a high prevalence population: The dai nationality, China. Genet. Med. 19 (9), 1022–1031. 10.1038/gim.2016.218 28125089

[B22] Huang J. Liang X. Xuan Y. Geng C. Li Y. Lu H. (2017). A reference human genome dataset of the BGISEQ-500 sequencer. Gigascience 6 (5), 1–9. 10.1093/gigascience/gix024 PMC5467036 28379488

[B23] Huang L. Ma F. Chapman A. Lu S. Xie X. S. (2015). Single-cell whole-genome amplification and sequencing: Methodology and applications. Annu. Rev. Genomics Hum. Genet. 16, 79–102. 10.1146/annurev-genom-090413-025352 26077818

[B24] Jiang F. Ren J. Chen F. Zhou Y. Xie J. Dan S. (2012). Noninvasive fetal trisomy (NIFTY) test: An advanced noninvasive prenatal diagnosis methodology for fetal autosomal and sex chromosomal aneuploidies. BMC Med. Genomics 5, 57. 10.1186/1755-8794-5-57 23198897 PMC3544640

[B25] Konstantinidis M. Prates R. Goodall N. N. Fischer J. Tecson V. Lemma T. (2015). Live births following karyomapping of human blastocysts: Experience from clinical application of the method. Reprod. Biomed. Online 31 (3), 394–403. 10.1016/j.rbmo.2015.05.018 26206283

[B26] Li G. Niu W. Jin H. Xu J. Song W. Guo Y. (2018). Importance of embryo aneuploidy screening in preimplantation genetic diagnosis for monogenic diseases using the karyomap gene chip. Sci. Rep. 8 (1), 3139. 10.1038/s41598-018-21094-6 29453426 PMC5816636

[B27] Li M. M. Datto M. Duncavage E. J. Kulkarni S. Lindeman N. I. Roy S. (2017). Standards and guidelines for the interpretation and reporting of sequence variants in cancer: A joint consensus recommendation of the association for molecular pathology, American society of clinical oncology, and college of American pathologists. J. Mol. Diagn. 19 (1), 4–23. 10.1016/j.jmoldx.2016.10.002 27993330 PMC5707196

[B28] Mai A. D. Harton G. L. Quang V. N. Van H. N. Thi N. H. Thuy N. P. (2020). Development and clinical application of a preimplantation genetic testing for monogenic disease (PGT-M) for beta thalassemia in Vietnam. J. Assist. Reprod. Genet. 38, 365–374. 10.1007/s10815-020-02006-y 33216308 PMC7884556

[B29] Mai A. D. Harton G. L. Quang V. N. Van H. N. Thi N. H. Thuy N. P. (2021). Development and clinical application of a preimplantation genetic testing for monogenic disease (PGT-M) for beta thalassemia in Vietnam. J. Assist. Reprod. Genet. 38 (2), 365–374. 10.1007/s10815-020-02006-y 33216308 PMC7884556

[B30] Masset H. Ding J. Dimitriadou E. Debrock S. Tsuiko O. Smits K. (2022). Single-cell genome-wide concurrent haplotyping and copy-number profiling through genotyping-by-sequencing. Nucleic Acids Res. 50, e63. 10.1093/nar/gkac134 35212381 PMC9226495

[B31] Masset H. Zamani Esteki M. Dimitriadou E. Dreesen J. Debrock S. Derhaag J. (2019). Multi-centre evaluation of a comprehensive preimplantation genetic test through haplotyping-by-sequencing. Hum. Reprod. 34 (8), 1608–1619. 10.1093/humrep/dez106 31348829

[B32] Sciorio R. Tramontano L. Catt J. (2020). Preimplantation genetic diagnosis (PGD) and genetic testing for aneuploidy (PGT-A): Status and future challenges. Gynecol. Endocrinol. 36 (1), 6–11. 10.1080/09513590.2019.1641194 31317806

[B33] Smith-Bindman R. Miglioretti D. (2015). Cell-free DNA analysis for noninvasive examination of trisomy. N. Engl. J. Med. 373 (26), 2581. 10.1056/NEJMc1509344 26699180

[B34] van Montfoort A. Carvalho F. Coonen E. Kokkali G. Moutou C. Rubio C. (2021). ESHRE PGT consortium data collection XIX-XX: PGT analyses from 2016 to 2017^†^. Hum. Reprod. Open 2021 (3), hoab024. 10.1093/hropen/hoab024 34322603 PMC8313404

[B35] Van Rij M. C. De Rademaeker M. Moutou C. Dreesen J. C. De Rycke M. Liebaers I. (2012). Preimplantation genetic diagnosis (PGD) for huntington's disease: The experience of three European centres. Eur. J. Hum. Genet. 20 (4), 368–375. 10.1038/ejhg.2011.202 22071896 PMC3306852

[B36] Vaz-de-Macedo C. Harper J. (2017). A closer look at expanded carrier screening from a PGD perspective. Hum. Reprod. 32 (10), 1951–1956. 10.1093/humrep/dex272 28938745

[B37] Wang Q. Xiang J. Sun J. Yang Y. Guan J. Wang D. (2019). Nationwide population genetic screening improves outcomes of newborn screening for hearing loss in China. Genet. Med. 21 (10), 2231–2238. 10.1038/s41436-019-0481-6 30890784

[B38] Wilton L. Thornhill A. Traeger-Synodinos J. Sermon K. D. Harper J. C. (2009). The causes of misdiagnosis and adverse outcomes in PGD. Hum. Reprod. 24 (5), 1221–1228. 10.1093/humrep/den488 19155287

[B39] Xu Y. Lin Z. Tang C. Tang Y. Cai Y. Zhong H. (2019). A new massively parallel nanoball sequencing platform for whole exome research. BMC Bioinforma. 20 (1), 153. 10.1186/s12859-019-2751-3 PMC6434795 30909888

[B40] Yan J. Qin Y. Zhao H. Sun Y. Gong F. Li R. (2021). Live birth with or without preimplantation genetic testing for aneuploidy. N. Engl. J. Med. 385 (22), 2047–2058. 10.1056/NEJMoa2103613 34818479

[B41] Yan L. Huang L. Xu L. Huang J. Ma F. Zhu X. (2015). Live births after simultaneous avoidance of monogenic diseases and chromosome abnormality by next-generation sequencing with linkage analyses. Proc. Natl. Acad. Sci. U. S. A. 112 (52), 15964–15969. 10.1073/pnas.1523297113 26712022 PMC4702982

[B42] Yang L. Wu Y. Hu Z. Zhang H. Pu D. Yan H. (2021). Simultaneous detection of fetal aneuploidy, de novo FGFR3 mutations and paternally derived beta-thalassemia by a novel method of noninvasive prenatal testing. Prenat. Diagn. 41 (4), 440–448. 10.1002/pd.5879 33340121 PMC8048498

[B43] Yang M. Rito T. Metzger J. Naftaly J. Soman R. Hu J. (2021). Depletion of aneuploid cells in human embryos and gastruloids. Nat. Cell Biol. 23 (4), 314–321. 10.1038/s41556-021-00660-7 33837289

[B44] Zhang C. Zhang C. Chen S. Yin X. Pan X. Lin G. (2013). A single cell level based method for copy number variation analysis by low coverage massively parallel sequencing. PLoS One 8 (1), e54236. 10.1371/journal.pone.0054236 23372689 PMC3553135

[B45] Zimmerman R. S. Jalas C. Tao X. Fedick A. M. Kim J. G. Pepe R. J. (2016). Development and validation of concurrent preimplantation genetic diagnosis for single gene disorders and comprehensive chromosomal aneuploidy screening without whole genome amplification. Fertil. Steril. 105 (2), 286–294. 10.1016/j.fertnstert.2015.10.003 26602983

