# Supplementary figures and images for: Simultaneous detection of genomic imbalance in patients receiving preimplantation genetic testing for monogenic diseases (PGT-M)
Source: Front Genet. 2022 Sep 29;13:976131. doi: 10.3389/fgene.2022.976131 (PMC9559864; doi:10.3389/fgene.2022.976131)

**A**

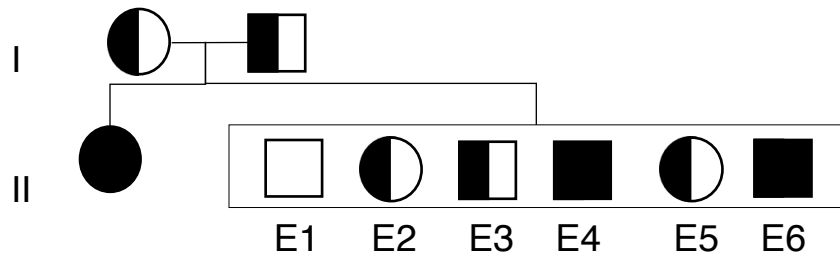

C

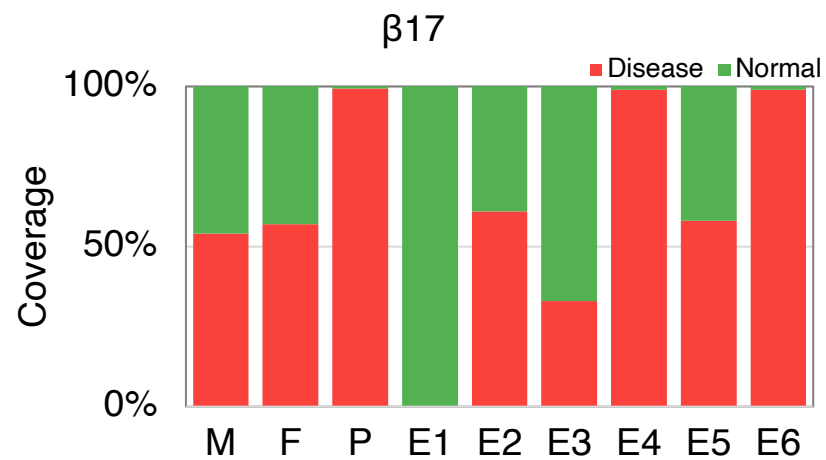

# B

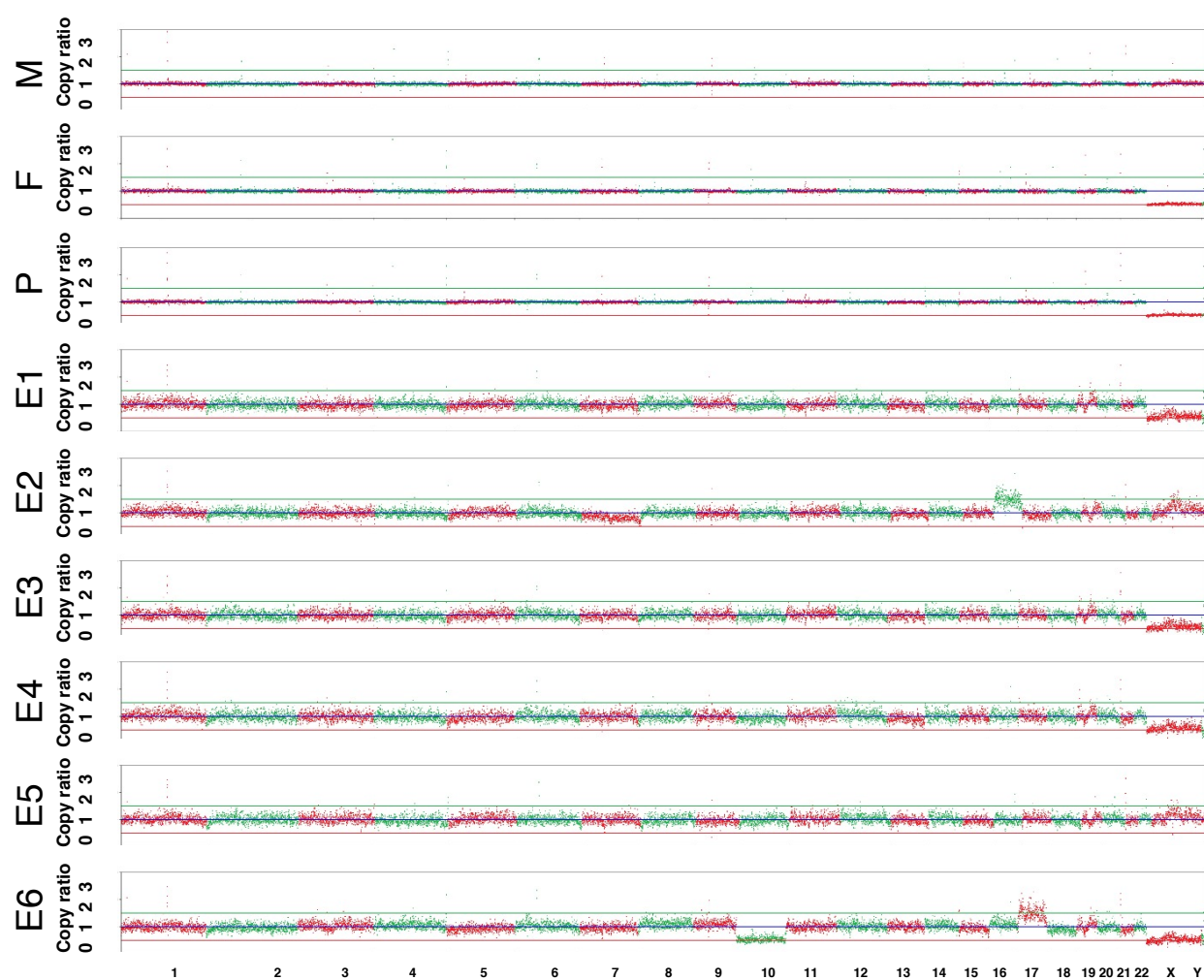

Supplement: Supplementary file 1 [file DataSheet7.PDF]

**A**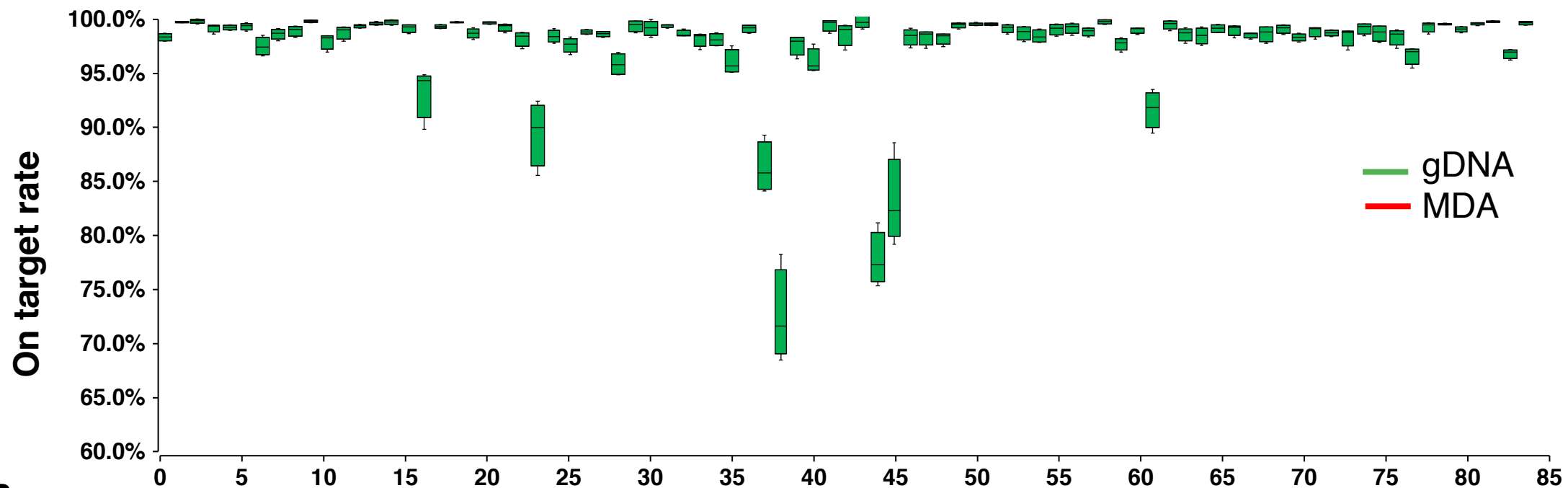**B**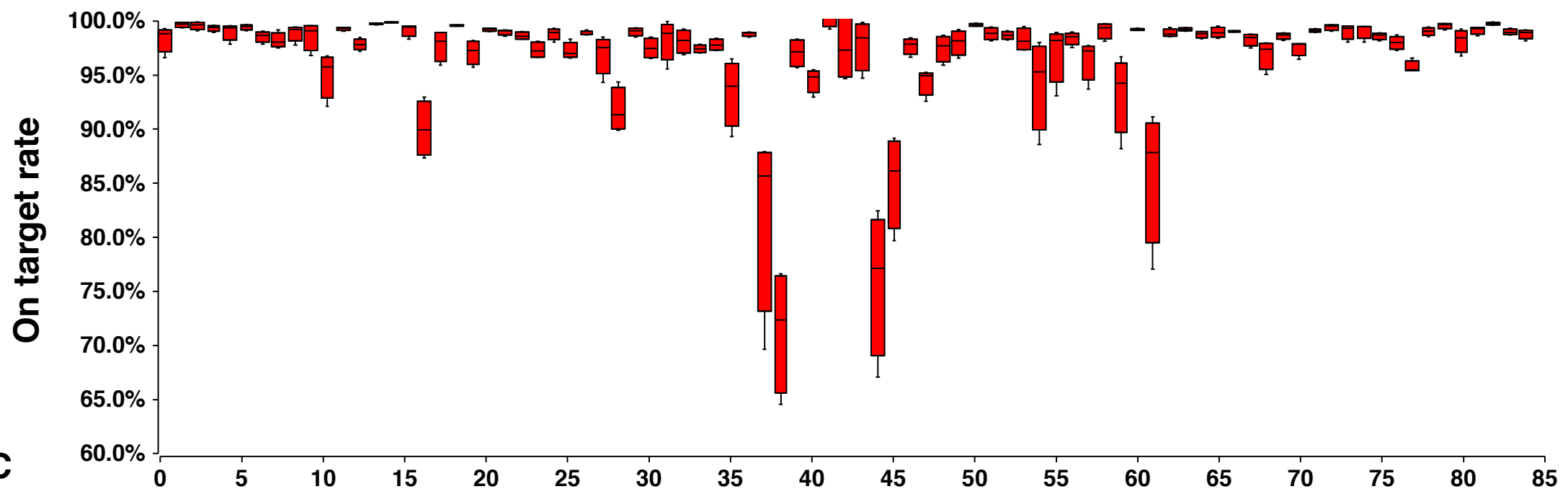**C**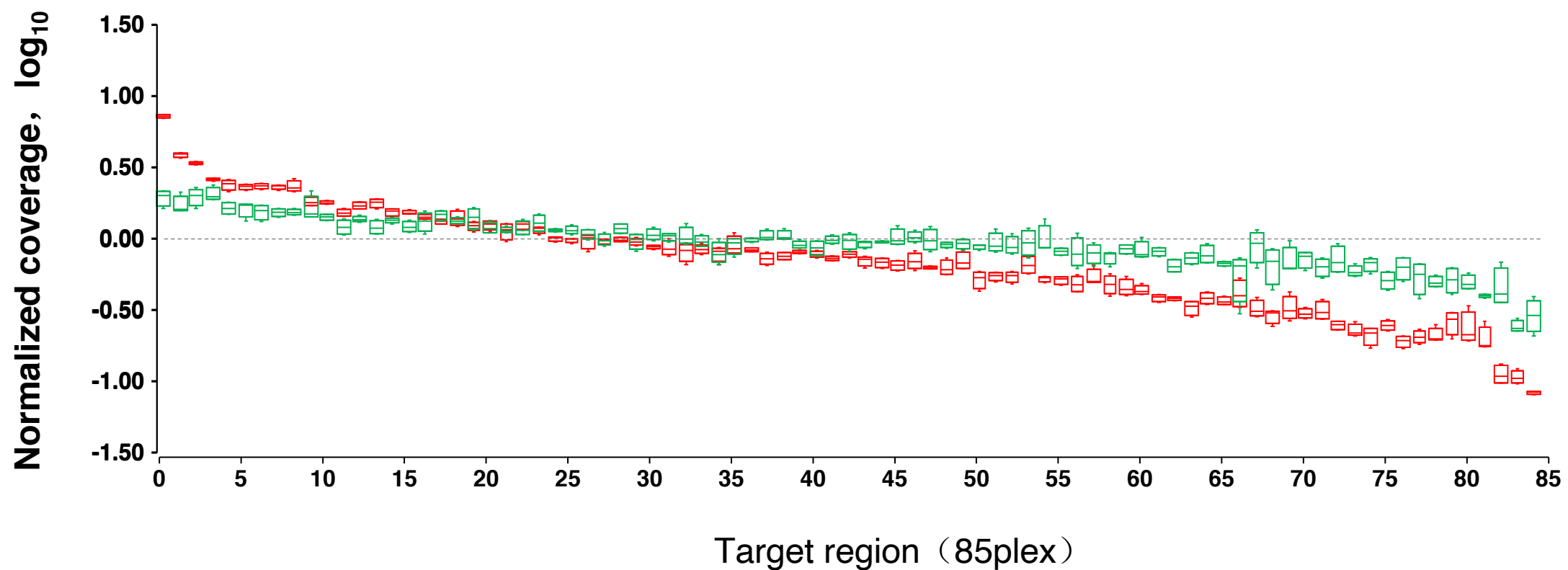

Supplement: Supplementary file 2 [file DataSheet2.PDF]

A

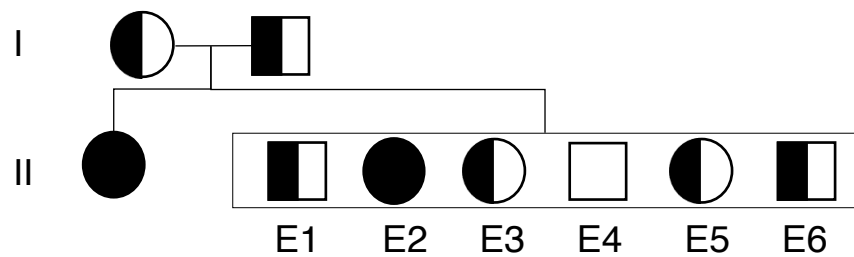

C

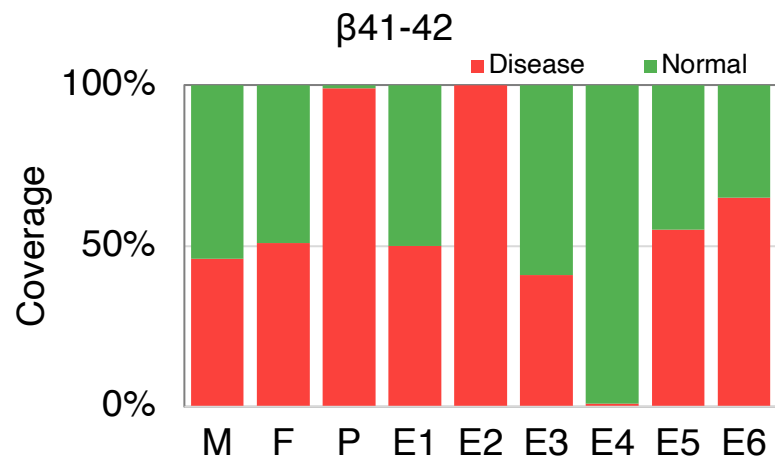

B

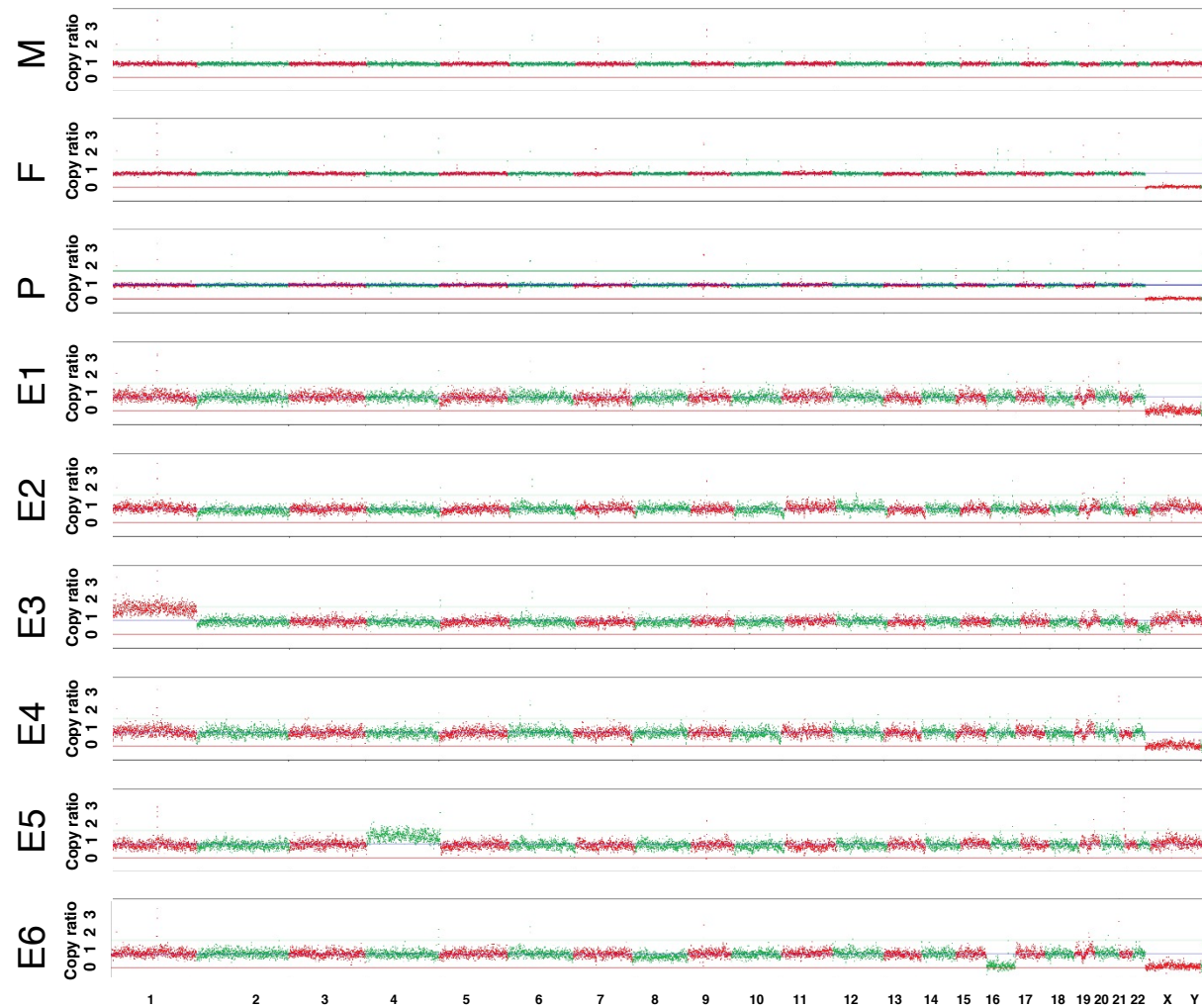

Supplement: Supplementary file 4 [file DataSheet4.PDF]

A

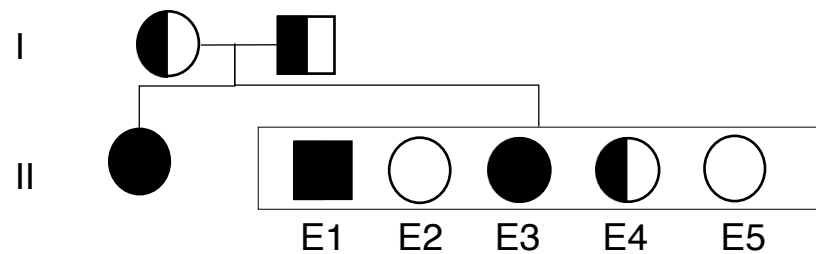

C

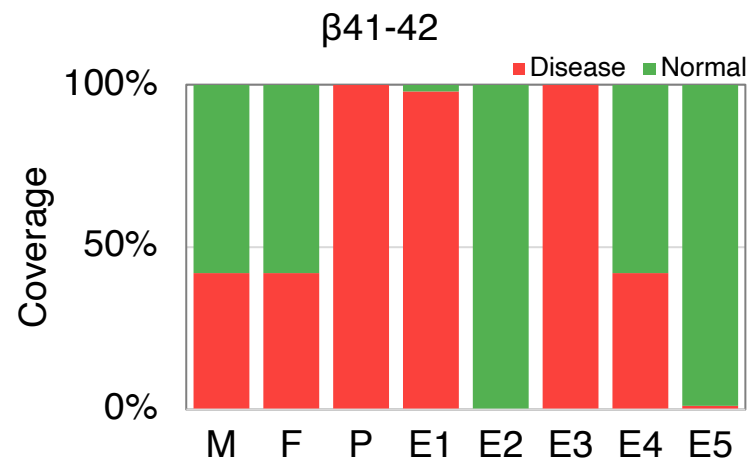

B

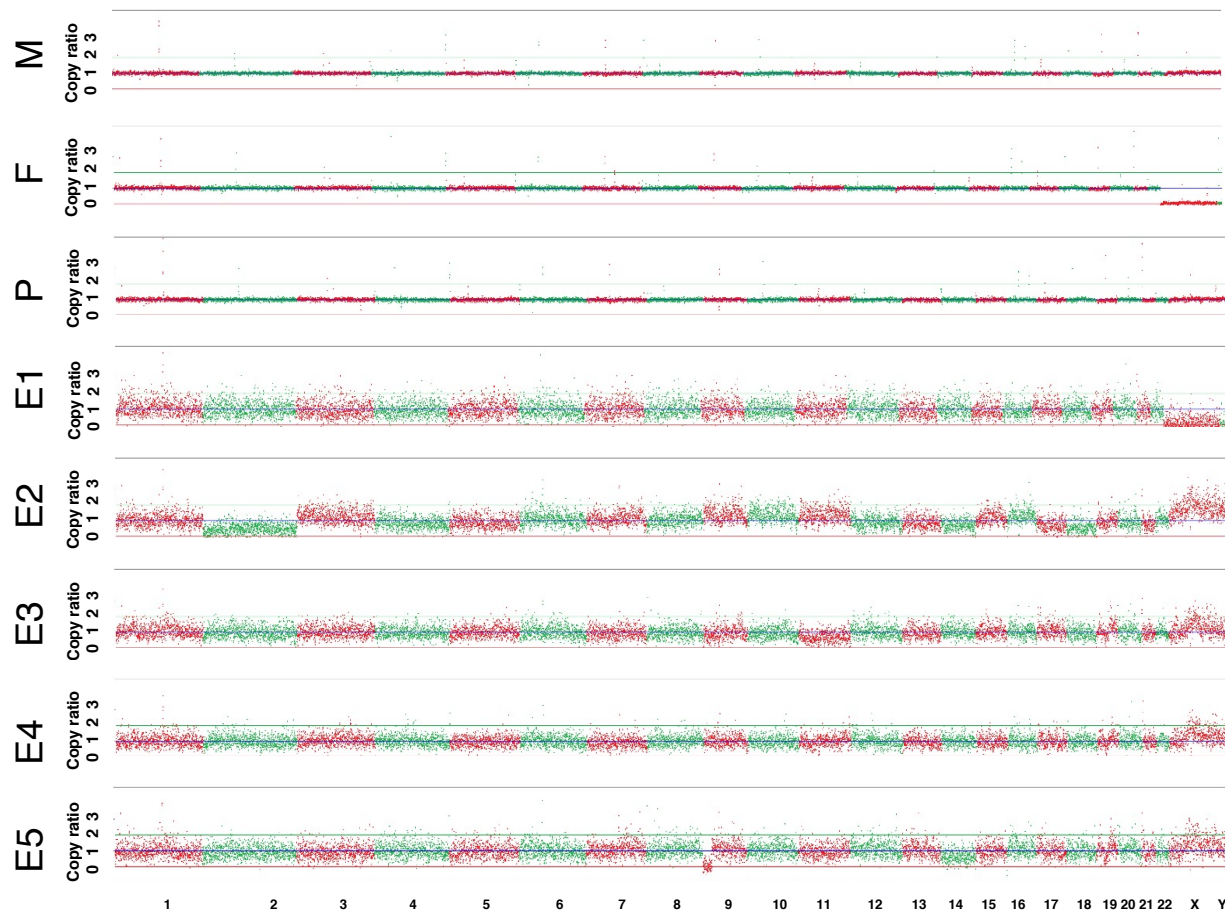

Supplement: Supplementary file 5 [file DataSheet6.PDF]

A

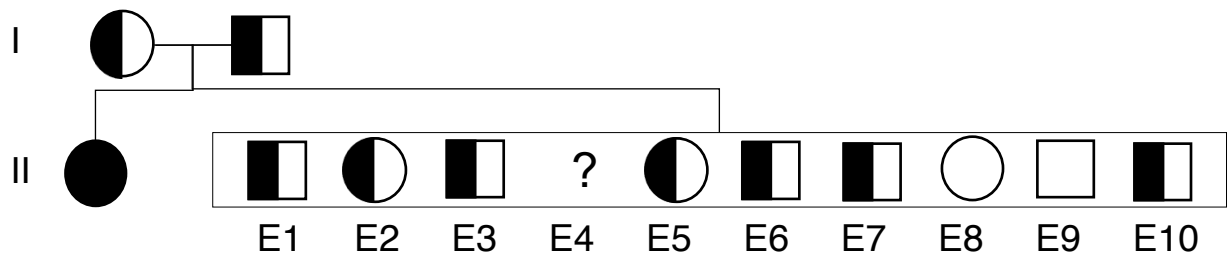

C

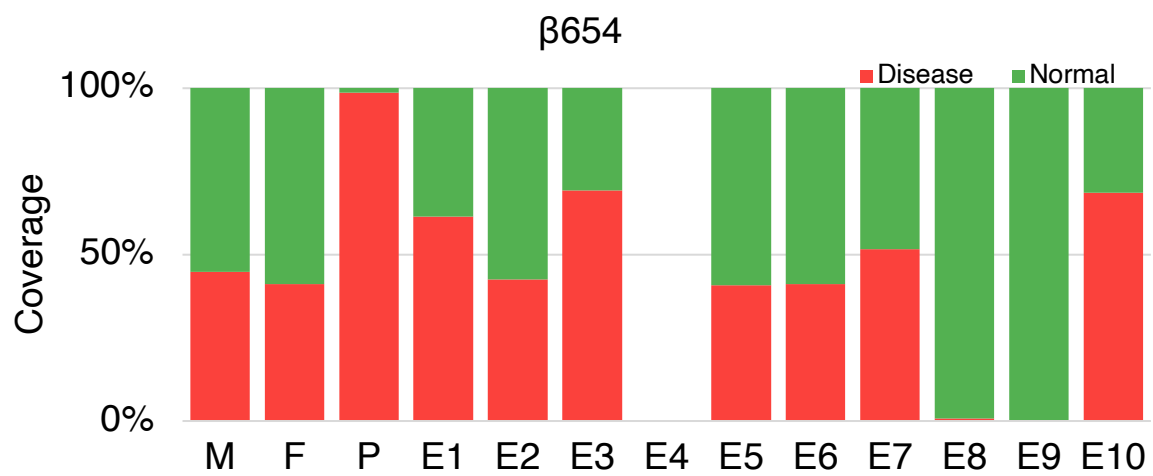

B

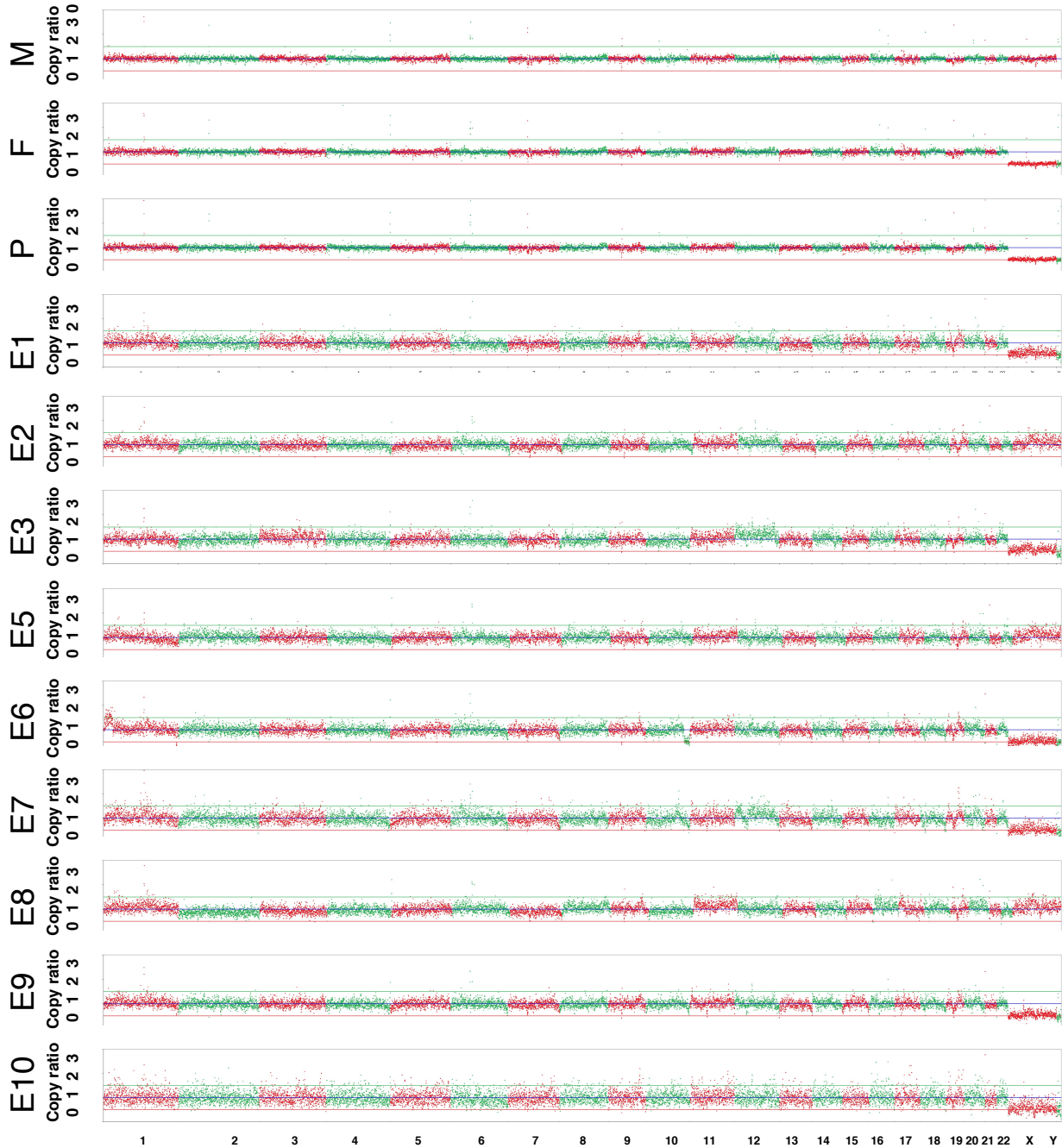

Supplement: Supplementary file 7 [file DataSheet9.PDF]

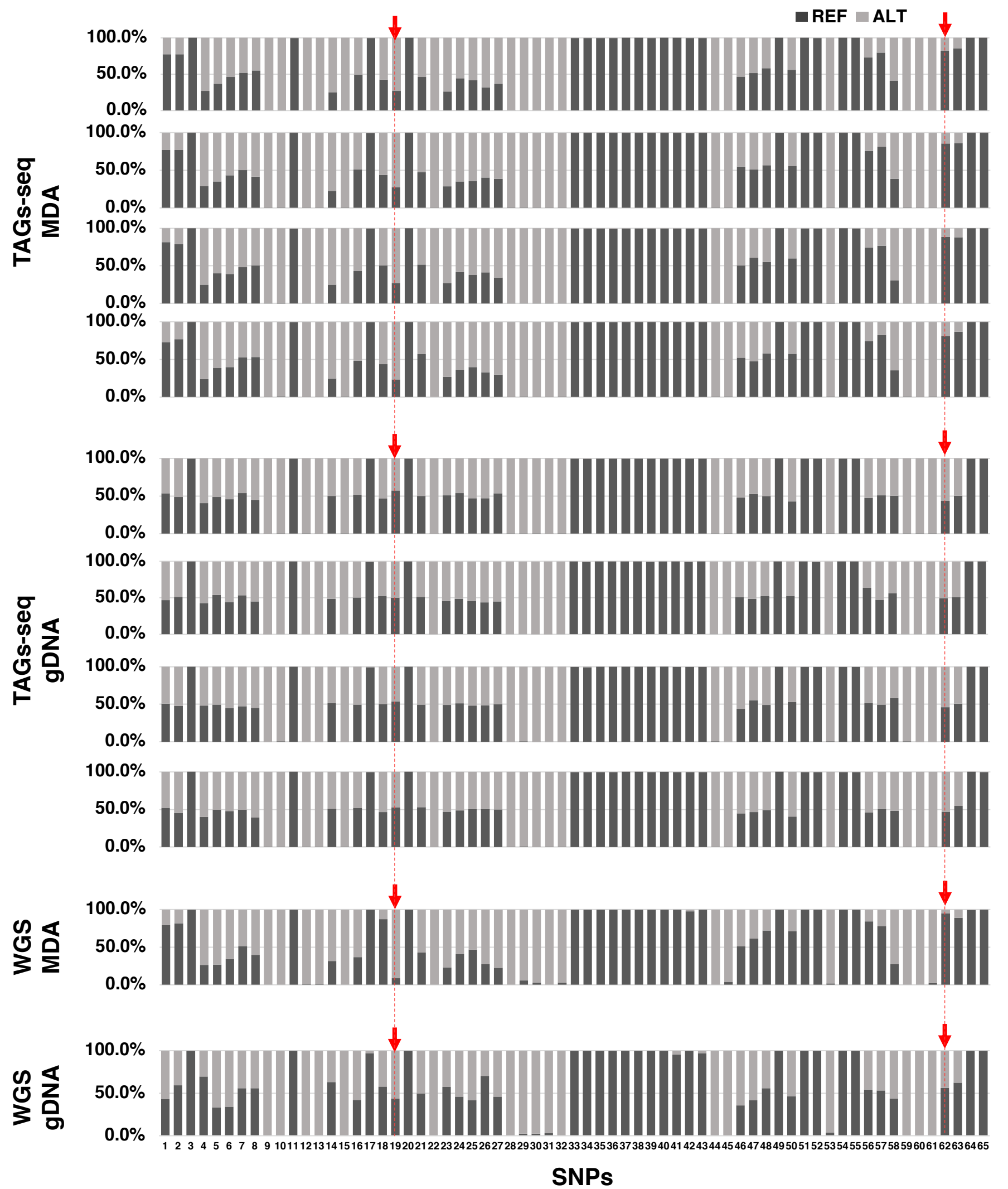

Supplement: Supplementary file 9 [file DataSheet3.PDF]

A

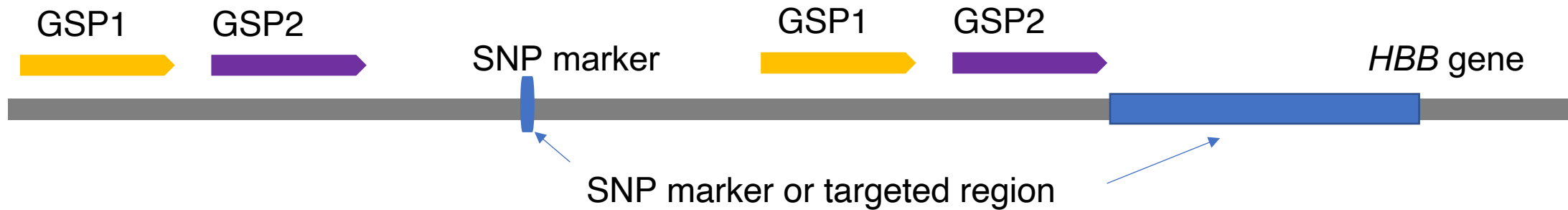

B

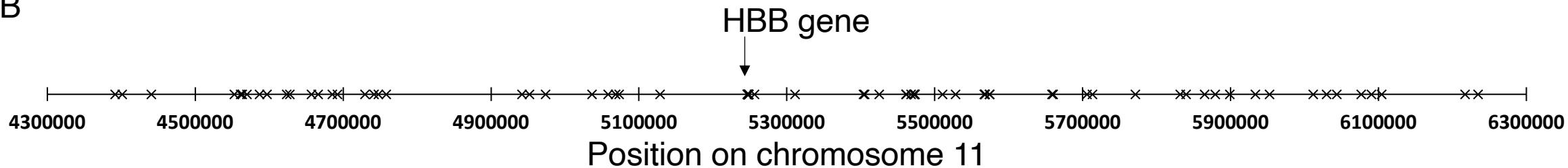

Supplement: Supplementary file 11 [file DataSheet1.PDF]

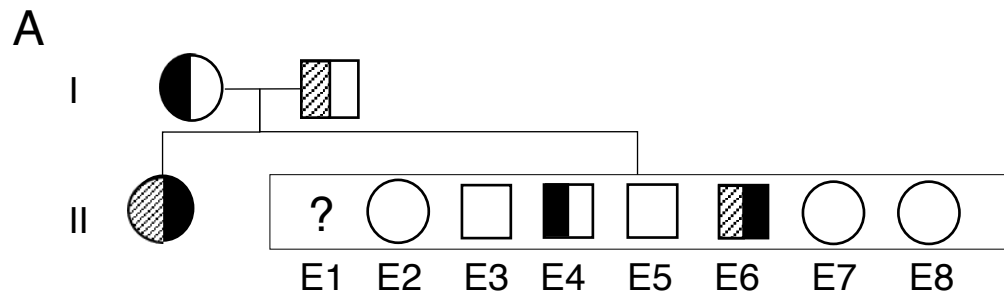

**B**

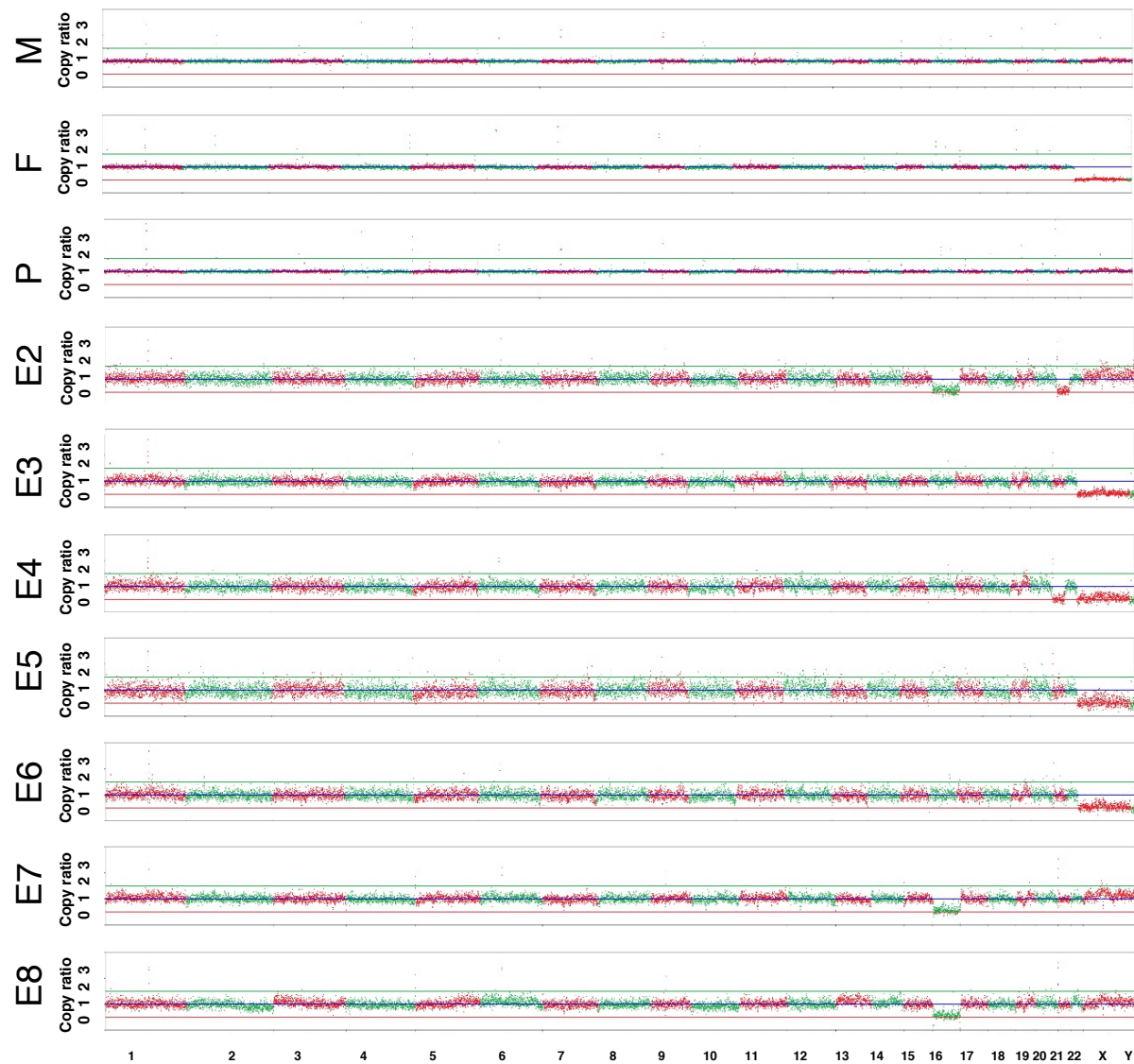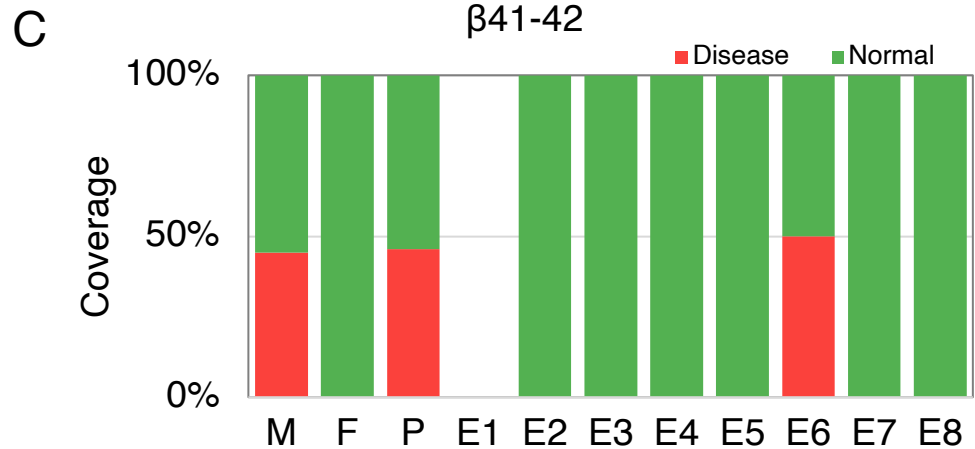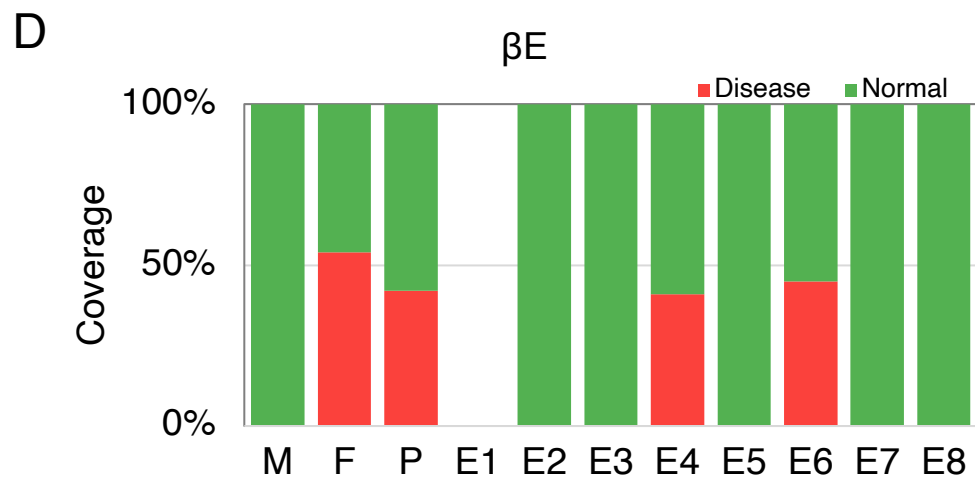

Supplement: Supplementary file 12 [file DataSheet5.PDF]

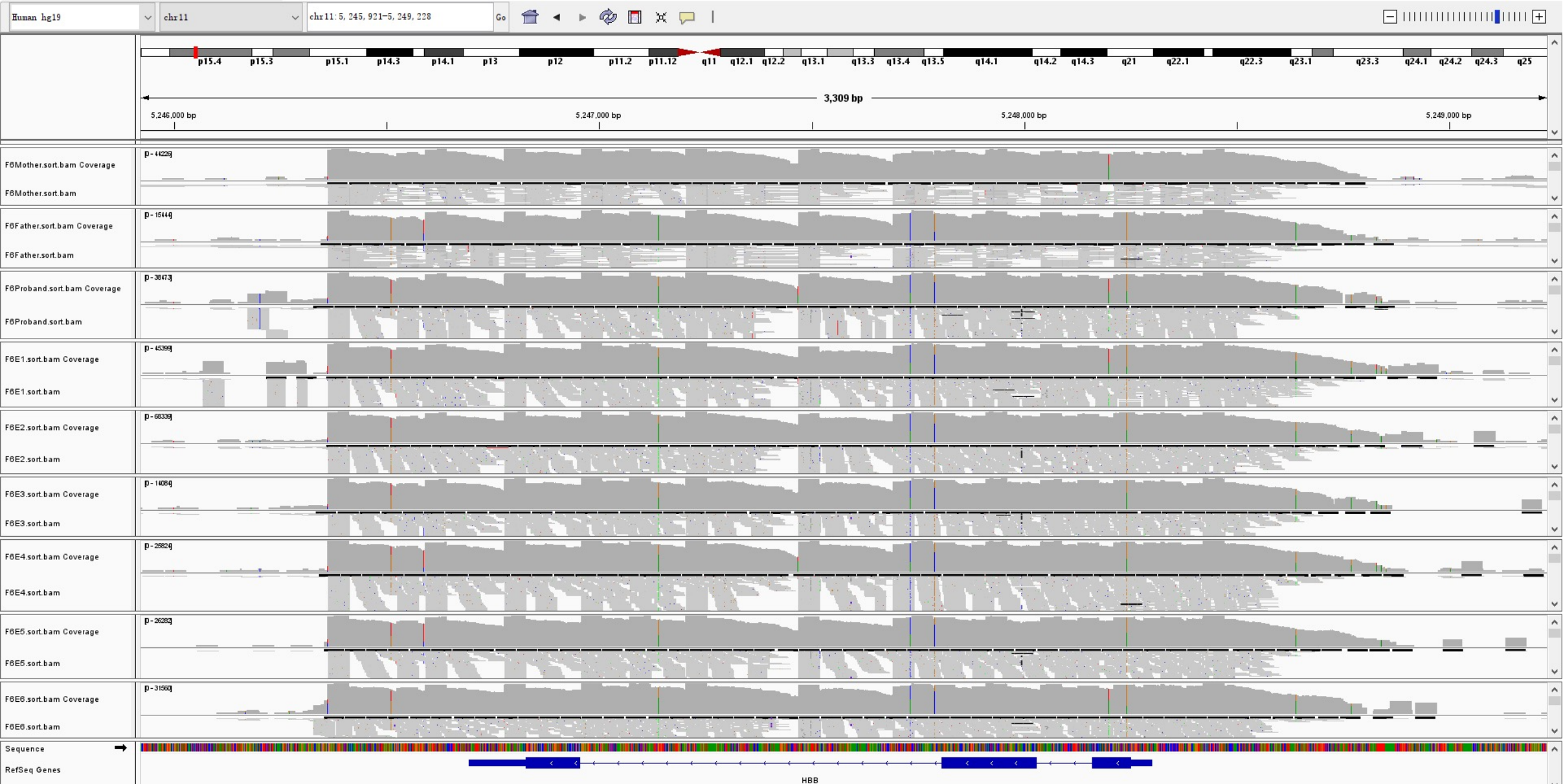

Supplement: Supplementary file 15 [file DataSheet12.PDF]

A

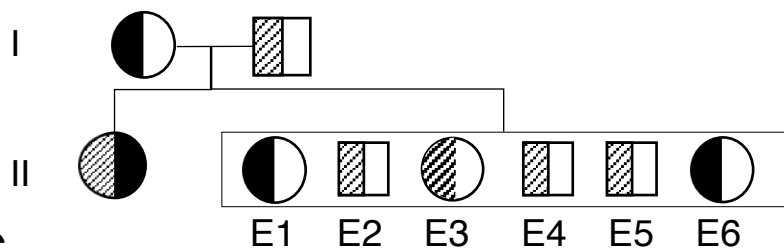

C

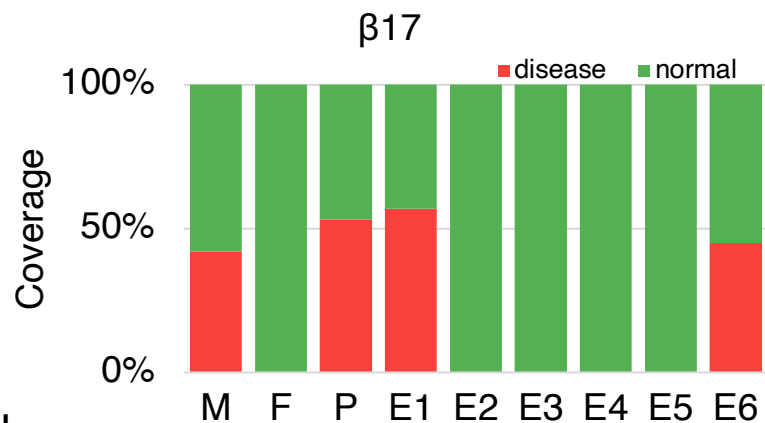

d

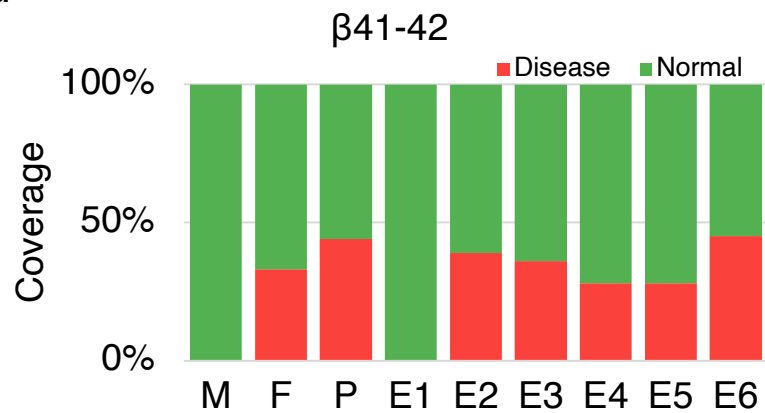

B

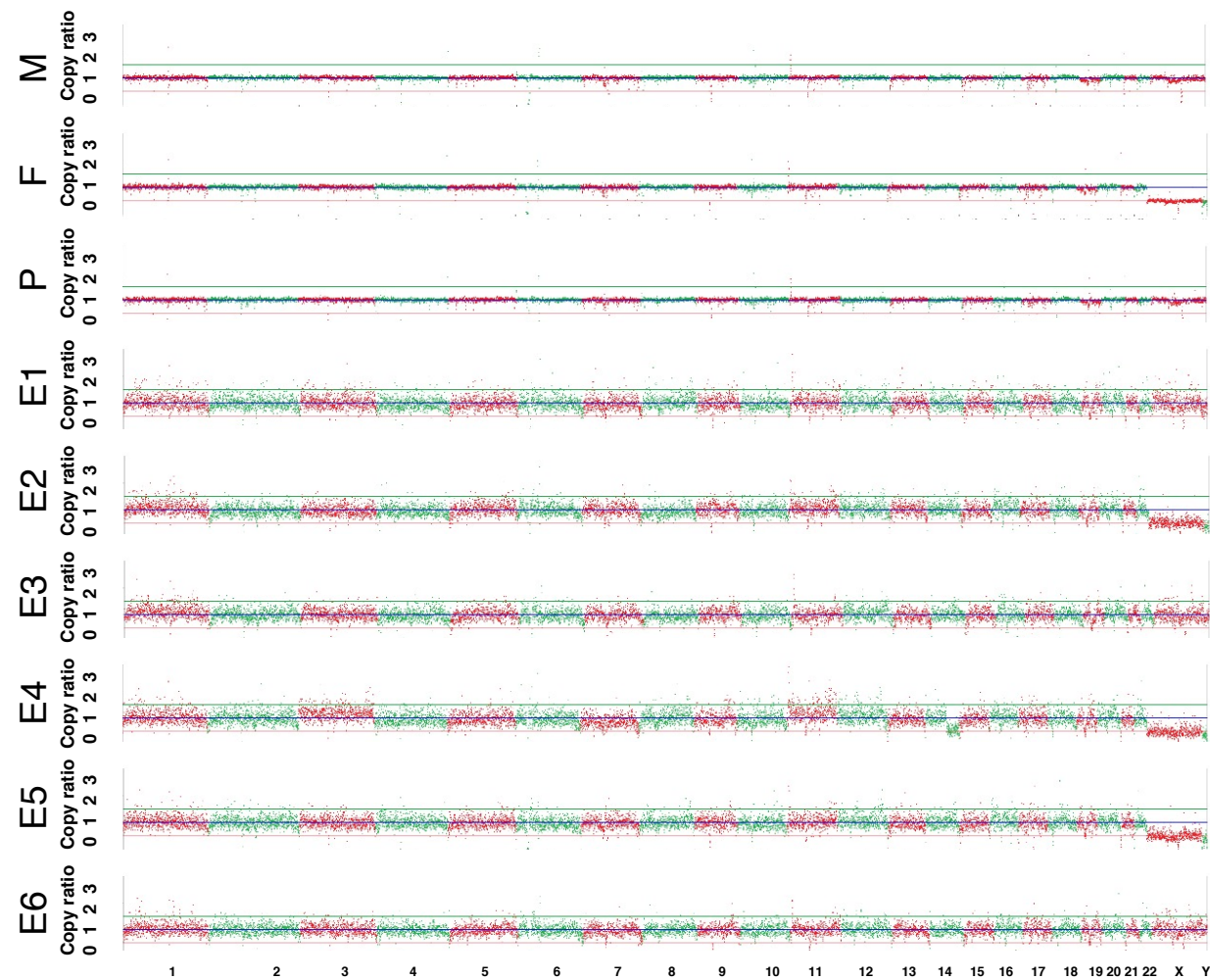

Supplement: Supplementary file 16 [file DataSheet8.PDF]

A

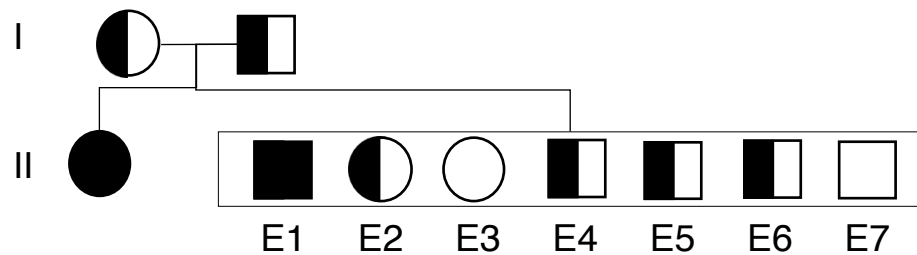

C

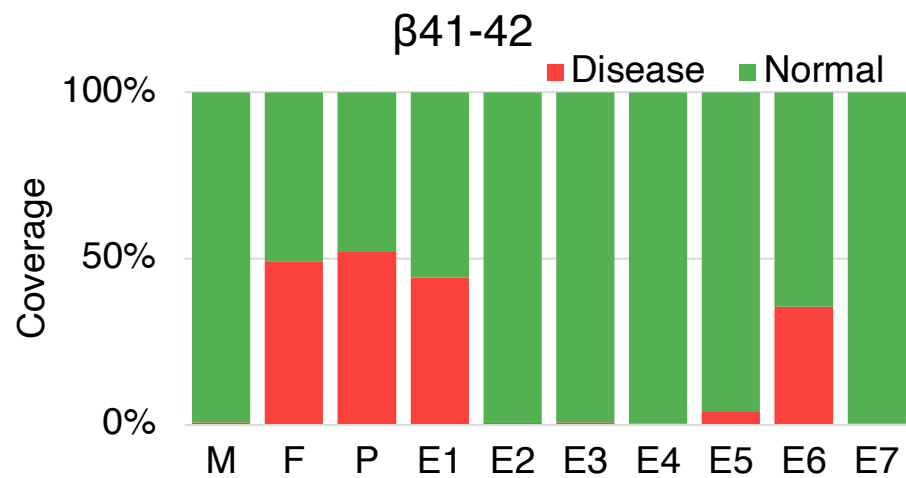

B

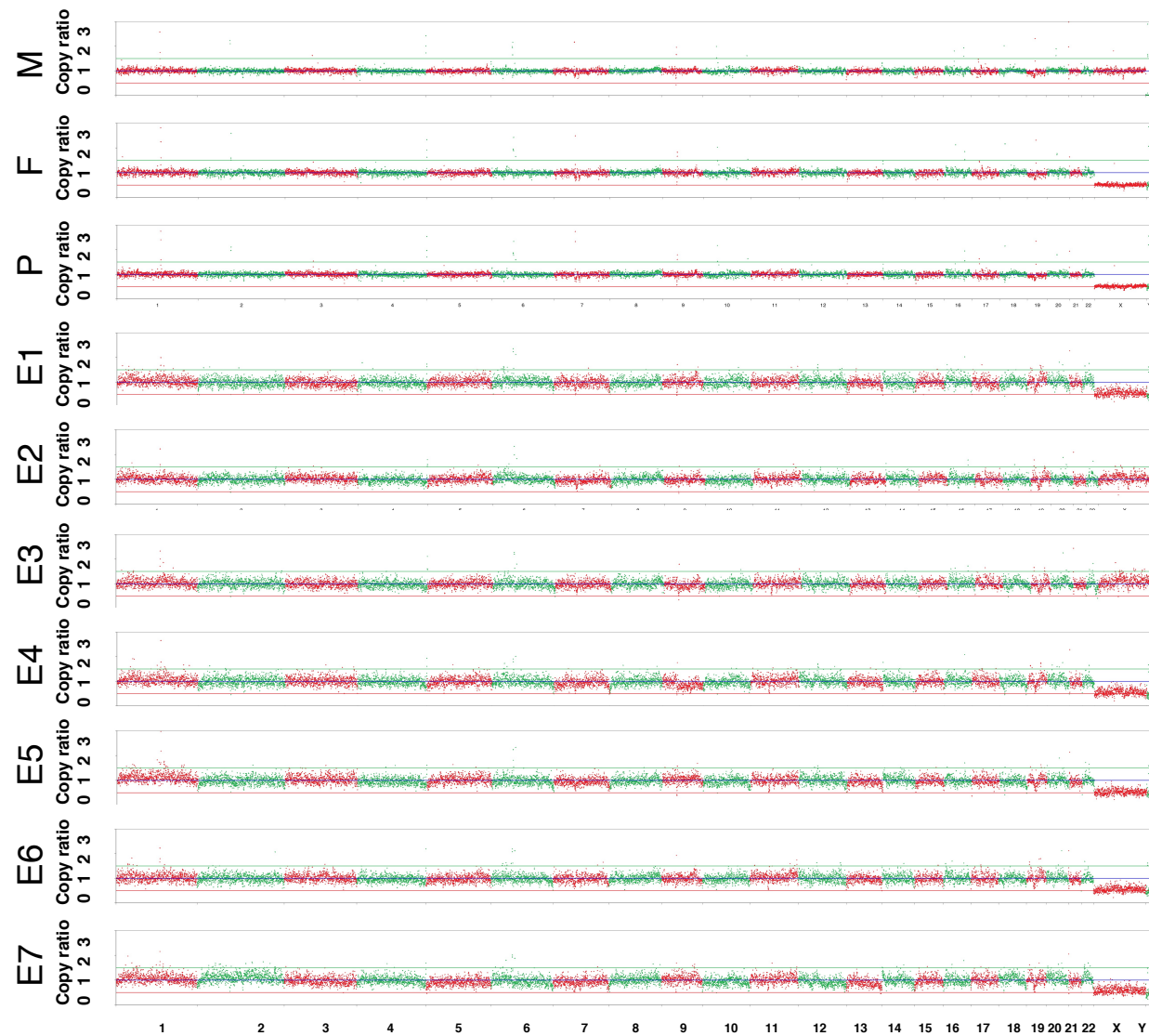

Supplement: Supplementary file 18 [file DataSheet10.PDF]
