# Supplementary material for: Simultaneous detection of genomic imbalance in patients receiving preimplantation genetic testing for monogenic diseases (PGT-M)
Source: Front Genet. 2022 Sep 29;13:976131. doi: 10.3389/fgene.2022.976131 (PMC9559864; doi:10.3389/fgene.2022.976131)
Supplement: Supplementary file 8 [file DataSheet11.PDF]

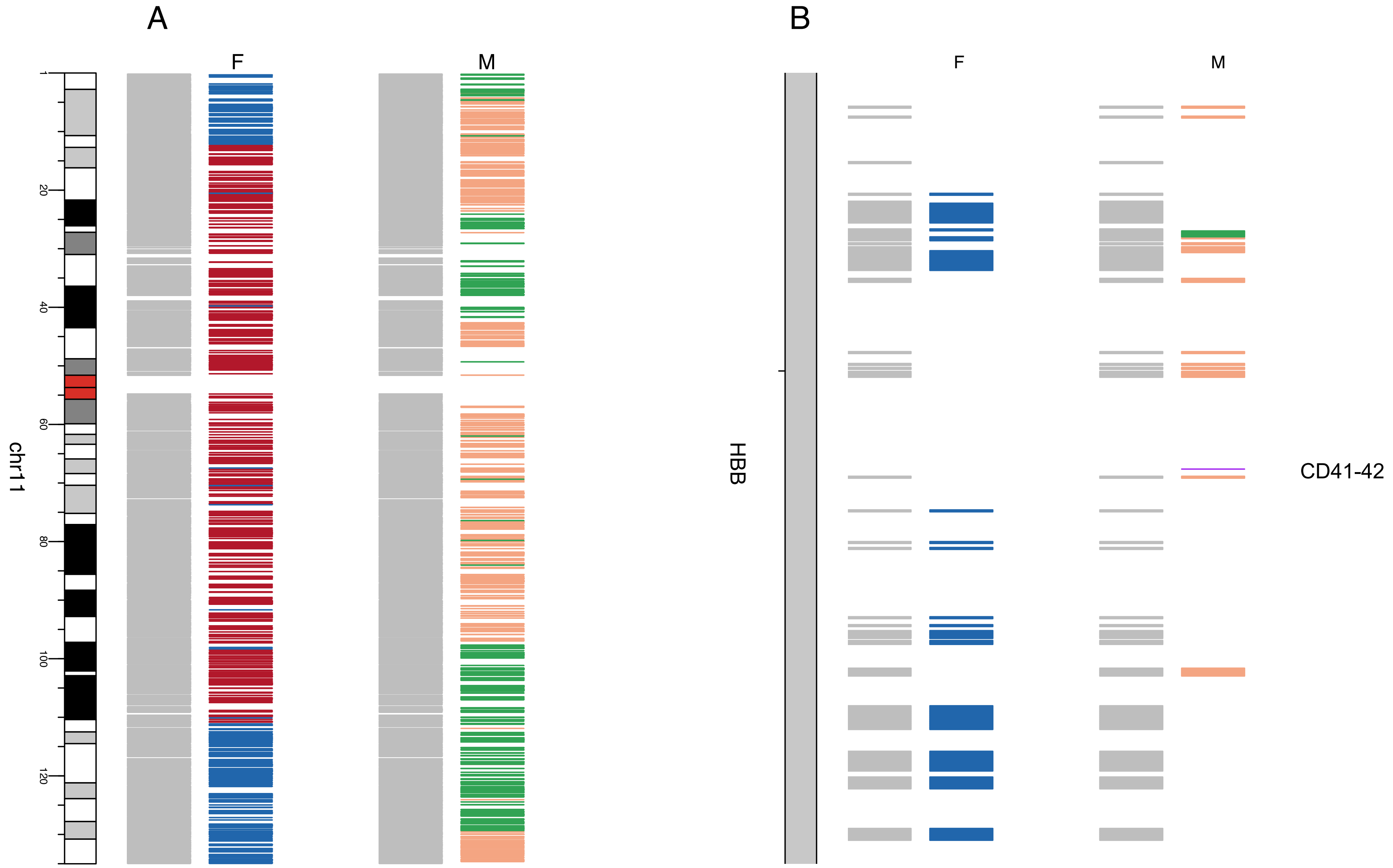
A
F
M
chr11
1
20
40
60
80
100
120
B
F
M
HBB
CD41-42
